# Utility of a PI3K/mTOR Inhibitor (NVP-BEZ235) for Thyroid Cancer Therapy

**DOI:** 10.1371/journal.pone.0046726

**Published:** 2012-10-15

**Authors:** Shu-Fu Lin, Yu-Yao Huang, Jen-Der Lin, Ting-Chao Chou, Chuen Hsueh, Richard J. Wong

**Affiliations:** 1 Department of Internal Medicine, Chang Gung Memorial Hospital, Chang Gung University, Taoyuan, Taiwan, Republic of China; 2 Department of Pathology, Chang Gung Memorial Hospital, Chang Gung University, Taoyuan, Taiwan, Republic of China; 3 Laboratory of Preclinical Pharmacology Core, Memorial Sloan-Kettering Cancer Center, New York, New York, United States of America; 4 Department of Surgery, Memorial Sloan-Kettering Cancer Center, New York, New York, United States of America; Complutense University, Spain

## Abstract

**Background:**

We assessed the utility of the dual PI3K/mTOR inhibitor NVP-BEZ235 (BEZ235) as single agent therapy and in combination with conventional chemotherapy for thyroid cancer.

**Methodology/Principal Findings:**

Eight cell lines from four types of thyroid cancer (papillary, follicular, anaplastic, medullary) were studied. The cytotoxicity of BEZ235 and five conventional chemotherapeutic agents alone and in combination was measured using LDH assay. Quantitative western blot assessed expression of proteins associated with cell cycle, apoptosis and signaling pathways. Cell cycle distribution and apoptosis were measured by flow cytometry. Murine flank anaplastic thyroid cancers (ATC) were treated with oral BEZ235 daily. We found that BEZ235 effectively inhibited cell proliferation of all cancer lines, with ATC exhibiting the greatest sensitivity. BEZ235 consistently inactivated signaling downstream of mTORC1. BEZ235 generally induced cell cycle arrest at G0/G1 phase, and also caused apoptosis in the most sensitive cell lines. Baseline levels of p-S6 ribosomal protein (Ser235/236) and p27 correlated with BEZ235 sensitivity. Growth of 8505C ATC xenograft tumors was inhibited with BEZ235, without any observed toxicity. Combination therapy of BEZ235 and paclitaxel consistently demonstrated synergistic effects against ATC *in vitro*.

**Conclusions:**

BEZ235 as a single therapeutic agent inhibits thyroid cancer proliferation and has synergistic effects in combination with paclitaxel in treating ATC. These findings encourage future clinical trials using BEZ235 for patients with this fatal disease.

## Introduction

Thyroid cancer is the most common endocrine malignancy, originating from thyroid follicular cells (papillary, follicular, poorly differentiated and anaplastic cancer) or parafollicular C cells (medullary cancer). The incidence of thyroid cancer has increased over the past 3 decades, primarily from an increase in the detection of papillary cancer. In contrast, the incidence of follicular and poorly differentiated thyroid cancer remains unchanged [1], [2]. Most patients with well-differentiated cancer, including papillary (PTC) and follicular (FTC) thyroid cancer have a favorable prognosis. Nevertheless, about 5% patients develop radioactive iodine refractory tumors and usually cause death within 5 years [3]. Anaplastic thyroid cancer (ATC) is a rare, highly aggressive, and often fatal disease, with a median survival of just 6 months. Medullary thyroid cancer (MTC) accounts for 3–5% of thyroid malignancy. The frequency of regional and distant metastatic disease in MTC diminishes survival rates [4], [5]. Novel therapies for refractory and aggressive thyroid cancer are needed to improve currently poor outcomes for these patients.

The PI3K/mTOR pathway is important for cell metabolism, survival and proliferation. Class I_A_ PI3Ks are heterodimers containing a p85 regulatory and a p110 catalytic subunits which phosphorylate phosphatidylinositol-4,5-biphosphate (PIP_2_), yielding phosphatidylinositol-3,4,5-triphosphate (PIP_3_). PIP_3_ combines with phosphoinositide-dependent protein kinase 1 (PDK1) to phosphorylate AKT at Thr308. In addition, the phosphorylation of AKT at Ser 473 by mTORC2 is required for full activity of AKT. Activation of AKT phosphorylates mTORC1, which subsequently phosphorylates S6 kinase1 and 4E-BP1, leading to G1/S cell cycle progression and inhibition of apoptosis. PTEN is a tumor suppressor that dephosphorylates PIP_3_ and inactivates this pathway [6], [7]. Alterations of this signaling pathway frequently occur in malignancies and are potential targets for cancer therapy [6]–[8].

In thyroid cancer, genetic alterations affecting the PI3K/mTOR pathway have been identified. *PIK3CA* (encoding p110α of class I_A_ PI3K) copy number gain correlates with increased *PIK3CA* protein expression. *PIK3CA* copy number gain occurs more frequently than genetic mutations of *PIK3CA* or *PTEN* in thyroid cancer [9]–[14]. More *PIK3CA/AKT1* mutations and *PIK3CA* copy gain are identified in ATC as compared to well differentiated cancer, suggesting that PI3K/mTOR pathway activity is involved in the process of cancer de-differentiation [3], [11], [12]. For MTC, *RET* proto-oncogene mutations occur in almost all familiar cases (25% of MTC) and about half of sporadic MTC. This gain-of-function rearrangement enhances PI3K/mTOR signaling transduction [15], [16]. In sporadic MTC without *RET* mutations, over 50% of tumor samples show activation of AKT or mTOR by immunohistochemistry [16].

BEZ235 is a dual PI3K/mTOR inhibitor that reduces PI3K and mTOR kinase activity by competitive binding to the ATP-binding cleft of these enzymes [17]. BEZ235 may treat cancers through induction of G0/G1 cell cycle arrest and apoptosis, and has recently entered phase II clinical trials [17]–[22]. This study was conducted to evaluate the efficacy of BEZ235 in treating thyroid cancer from four major pathological types, including PTC, FTC, ATC and MTC. We also explored combination effects of BEZ235 and currently employed chemotherapeutics against four ATC cell lines.

## Results

### Cytotoxicity of BEZ235

**Figure 1:**
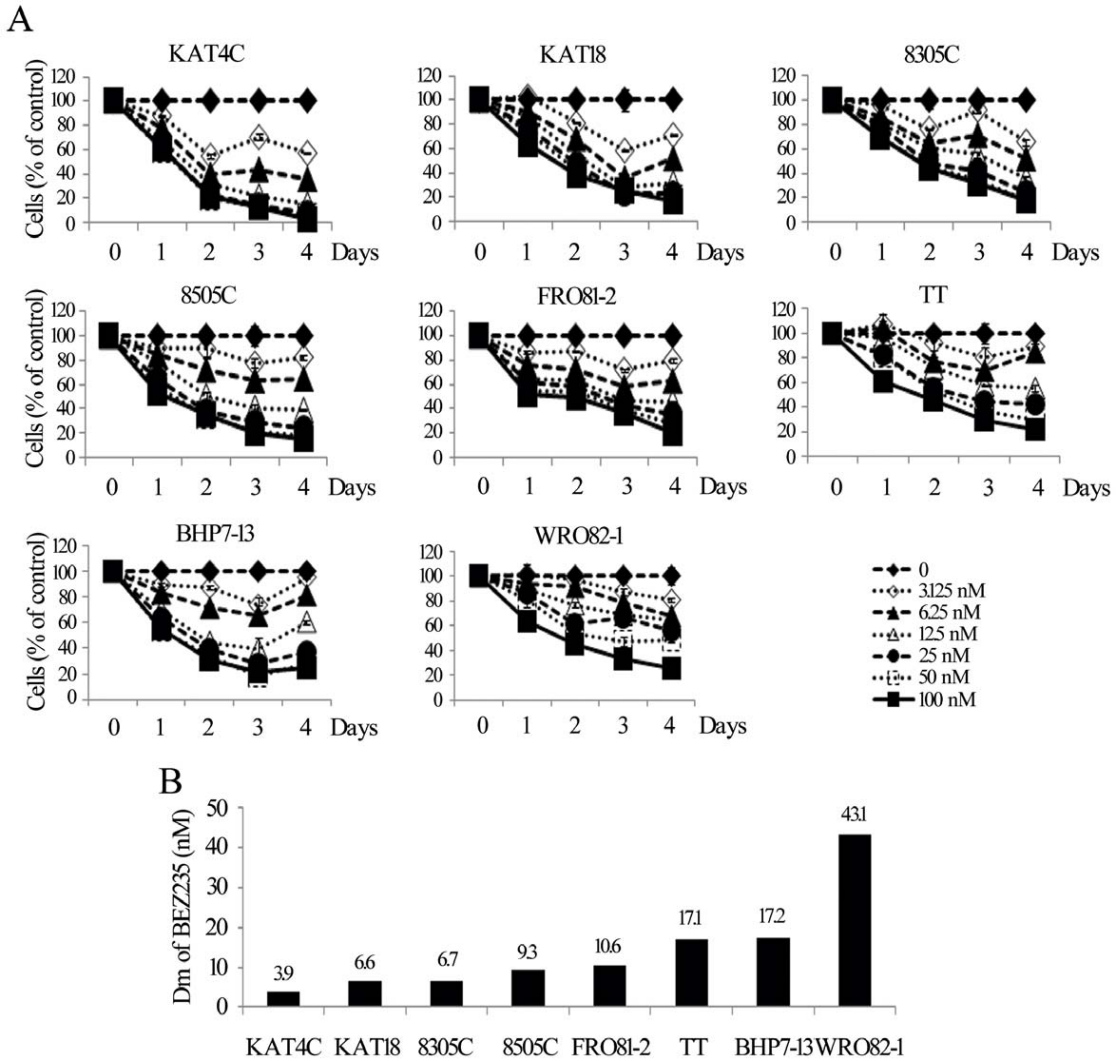
BEZ235 induces dose and time dependent cytotoxicity in 8 thyroid cancer cell lines. A, dose-response curves were obtained daily from cells treated with a range of six 1∶1 dilutions of BEZ235. B, the Dm of BEZ235 on day 4 was calculated using CompuSyn software for each cell line. ATC cell lines (KAT4C, KAT18, 8305C and 8505C) had the lowest Dm and thus most sensitive, follow by follicular undifferentiated thyroid cancer (FRO81-2), medullary (TT) and well-differentiated papillary (BHP7-13) and follicular (WRO82-1) cancer.

BEZ235 inhibited cell proliferation in all thyroid cancer lines in a dose and time dependent manner (Figure 1A). A low dose of BEZ235 at 6.25 nmol/L impeded at least 30% of cell growth in 6 of 8 cell lines on day 4. BEZ235 at 100 nmol/L arrested more than 80% cell growth in ATC lines, 78% in medullary (TT) and more than 74% in well-differentiated thyroid cancer lines. The Dm of BEZ235 on day 4 was calculated for each cell line (Figure 1B). The 4 ATC lines were the most sensitive to BEZ235 (Dm, KAT4C = 3.9, KAT18 = 6.6, 8305C = 6.7, 8505C = 9.3 nmol/L), followed by the follicular undifferentiated cancer FRO81–2 (Dm, 10.6 nmol/L). The medullary thyroid cancer (TT), the well-differentiated papillary (BHP7-13), and the follicular (WRO82–1) cancer were less sensitive (Dm 17.1, 17.2, and 43.1 nmol/L, respectively).

### Modulation of signaling pathways by BEZ235

**Figure 2:**
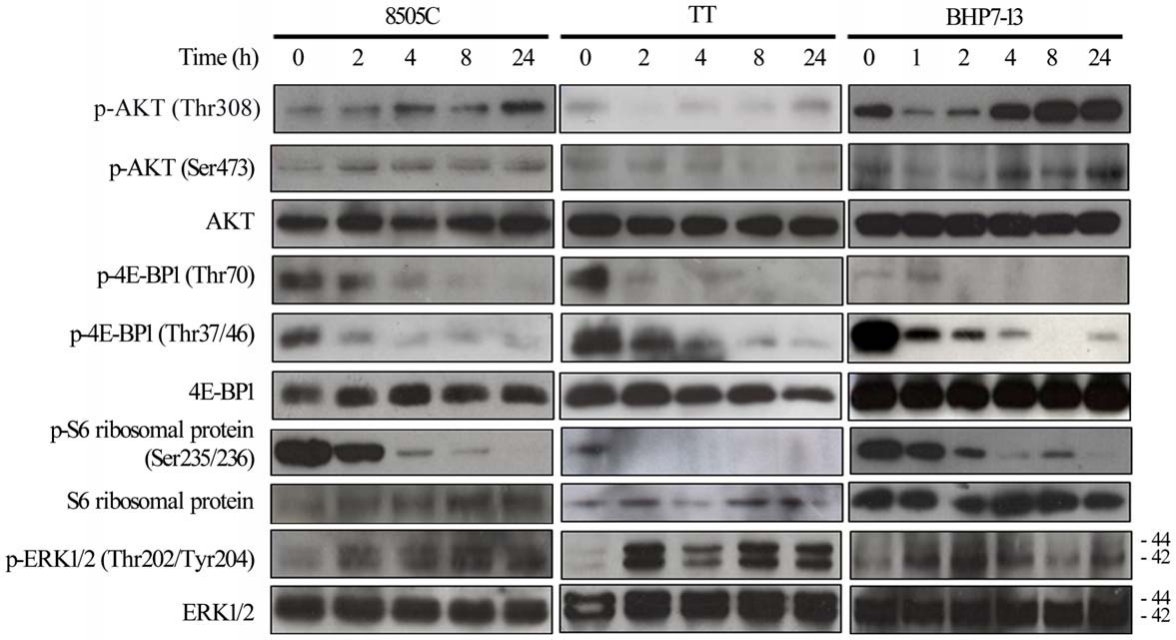
mTORC1downstream signaling is inhibited and p-ERK1/2 is activated by BEZ235 at 100 nmol/L. p-AKT (Thr308) and p-AKT (Ser473) was increased in 8505C for more than 24 hours. p-AKT (Thr308) and p-AKT (Ser473) was decreased transiently in TT and BHP7-13. p-4E-BP1 (Thr70), p-4E-BP1 (Thr37/46) and p-S6 ribosomal protein (Ser235/236) were consistently reduced from 2 hours through over 24 hours in three cell lines. BEZ235 caused a rapid increase of p- ERK1/2 (Thr202/Tyr204) by 2 to 4 hours in three cell lines.

The effects of BEZ235 on signaling pathways were examined in 8505C, TT and BHP7-13 cell lines (Figure 2, Figure S1). p-4E-BP1 (Thr70), p-4E-BP1 (Thr37/46) and p-S6 ribosomal protein (Ser235/236) were all consistently repressed by BEZ235 within 2 hours and the inhibition effect was significant and durable, with less than 12% of protein detected at 24 hours. p-ERK1/2 (Thr 202/Tyr204) was activated by BEZ235 with 2.6 to 8.6-fold increase at 2 to 8 hours in three cell lines. However, BEZ235 had heterogeneous effects on p-AKT (Thr308) and p-AKT (Ser473), which could be inhibited for a period in TT and BHP7-13, but were persistently increased in 8505C. As in 8505C cells, in KAT4C cells BEZ235 similarly inhibited p-S6 ribosomal protein (Ser235/236), but activated p-AKT (Thr308), p-AKT (Ser473) and p-ERK1/2 (Thr202/Tyr204), (Figure S1 and S2A). The immunoblot was quantified and statistical analysis was performed for p-4E-BP1 (Thr37/46) in BHP7-13 and p-S6 ribosomal protein (Ser235/236) in 8505C and BHP7-13. BEZ235 profoundly inhibited these proteins by 2 to 4 hours, and this effect was durable for 24 hours (Figure S1). p-4E-BP1 (Thr37/46) was repressed to less than 12% from 2 to 24 hours and achieved statistical significance when compared with basal level in BHP7-13 (P<0.02, t-test). Similarly, p-S6 ribosomal protein (Ser235/236) was significantly decreased to less than 7% and 13% from 4 to 24 hours in 8505C (P<0.01, t-test) and BHP7-13 (P<0.02, t-test), respectively. These results suggest that BEZ235 has a strong ability to inhibit mTORC1 (leading to decreases of p-4E-BP1 and p-S6 ribosomal protein) and weaker effects on mTORC2 (leading to varied effects on p-AKT at Ser473).

### Effects of BEZ235 on cell cycle and apoptosis

**Figure 3:**
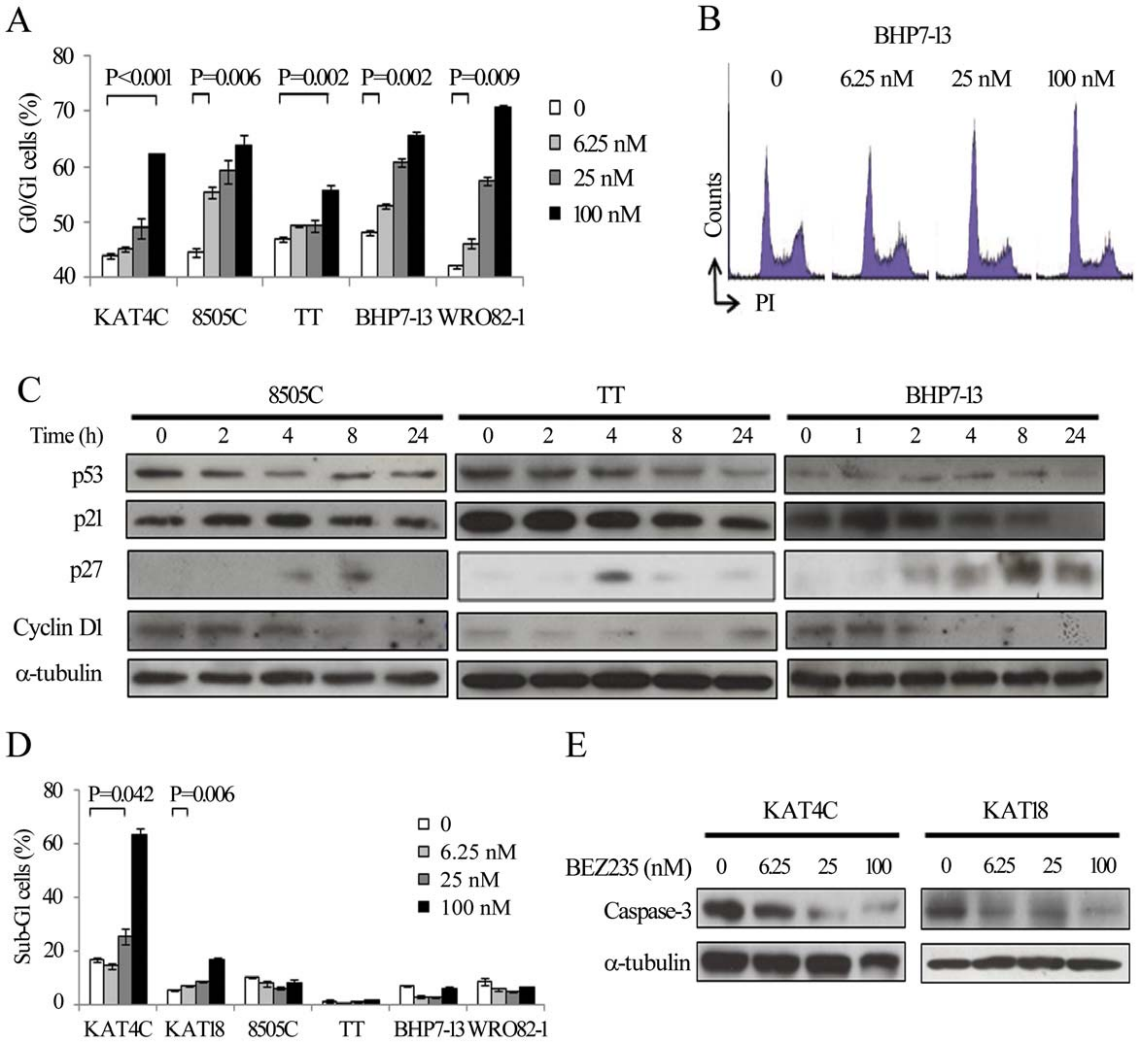
BEZ235 inhibits cell cycle progression at G0/G1 phase, and induces apoptosis in KAT4C and KAT18. A, analysis of cell cycle by measurements of DNA content revealed increasing doses of BEZ235 accumulates cells at G0/G1 by 24 hours (KAT4C, 8505C, BHP7-13 and WRO82-1) and 72 hours (TT). B, a representative cell line, BHP7-13 demonstrated cell cycle arrest at G0/G1 phase. C, cell cycle regulating proteins were evaluated using immunoblot in cells treated with BEZ235 at 100 nmol/L. p53 was decreased by 2 to 4 hours in 8505C and TT. BEZ235 gradually reduced p21 in TT and BHP7-13. p27 was increased in all cell lines. Cyclin D1 gradually decreased in 8505C and BHP7-13. D, apoptosis measured by flow cytometry to measure sub-G1 cells revealed that BEZ235 increases proportions of sub-G1 cells in KAT4C and KAT18 at 96 hours. E, immunoblot showed BEZ235 induced degradation of executioner caspase-3 in KAT4C and KAT18.

KAT4C, 8505C, TT, BHP7-13 and WRO82-1 were exposed to BEZ235 for 24 or 72 hours (Figure 3A). Compared with untreated control cells, BEZ235 at 6.25, 25 and 100 nmol/L induced increasing cell fractions at G0/G1phase in all cell lines. A representative cell line BHP7-13 demonstrated the effect of BEZ235 on cell cycle distribution (Figure 3B).

The effects of BEZ235 (100 nmol/L) on the expression of G0/G1 related cell cycle proteins was examined (Figure 3C, Figure S2B and S3). The transition of G0/G1 to S phase is a complex process involving cyclins, cyclin-dependent kinases (CDKs) and associated proteins, including p53, p21, p27 and cyclin D1 [31]. p53 is a tumor suppressor that may activate p21, and both p21 and p27 are inhibitors of G1 cyclin-dependent kinases.

BEZ235 increased p27 by 4 to 24 hours in all cell lines, with maximal increases of 3.5 to 723.2- fold in KAT4C, TT and BHP7-13. Such a ratio of increase was not measurable in 8505C due to undetectable signal in untreated cells. Statistical analysis was performed for p27 expression in TT, and achieved significance at 8 hours as compared with basal expression (P = 0.019, t-test) (Figure S3C). This data is consistent with our finding of G0/G1 cell cycle arrest. BEZ235 had varied effects on the expression of p53, p21 and cyclin D1 in 4 cell lines, showing that there may be varied contributions of these proteins on affecting cell cycle progression. A consistent increase of p27 in BEZ235 treated cells likely exerted inhibitory effects on cell cycle progression at G0/G1. It is possible that G0/G1 cell cycle arrest is the result of a combined effect of more than one G0/G1 related protein affected by BEZ235. Alternatively, the induction of p27 alone may be enough to arrest cell cycle progression. In a p53 negative PC3M cell line, BEZ235 induced p27 expression and complete cell cycle arrest at G1 phase, without inducing p21 [17]. This study demonstrated G1 cell cycle inhibition induced by BEZ235 does not necessary require association with p53 and p21.

The inhibition of the PI3K/mTOR pathway may also lead to apoptosis [6], [8]. The ability of BEZ235 to cause apoptotic cell death in thyroid cancer cells was explored (Figure 3D). Compared with control, BEZ235 at 25 and 100 nmol/L significantly induced apoptosis as measured by the proportion of sub-G1 cells at 96 hours in KAT4C. Similar findings were observed in KAT18, with BEZ235 at 6.25, 25 and 100 nmol/L driving an increasing proportion of apoptotic cells. However, BEZ235 failed to show any increase of sub-G1 cells in 8505C, TT, BHP7-13, and WRO82-1.

To validate the induction of apoptosis in KAT4C and KAT18 by BEZ235, caspase-3 was assessed by immunoblot after 72 hours of treatment (Figure 3E, Figure S4). In general, higher doses of BEZ235 induced more degradation of apoptotic executioner caspase-3, with less than 12% of caspase-3 detected at 100 nmol/L in both cell lines. This data suggests that apoptotic mechanisms account for the cytotoxicity of BEZ235 in KAT4C and KAT18. These findings are consistent with previous reports of BEZ235 causing apoptosis in some, but not all, cell lines [17]–[22].The underlying mechanisms of the varied abilities of BEZ235 to induce apoptosis at different doses and in different cell lines remain unclear [6]–[8].

### BEZ235 sensitivity correlates with baseline expression of p-S6 ribosomal protein (Ser235/236) and p27

**Figure 4:**
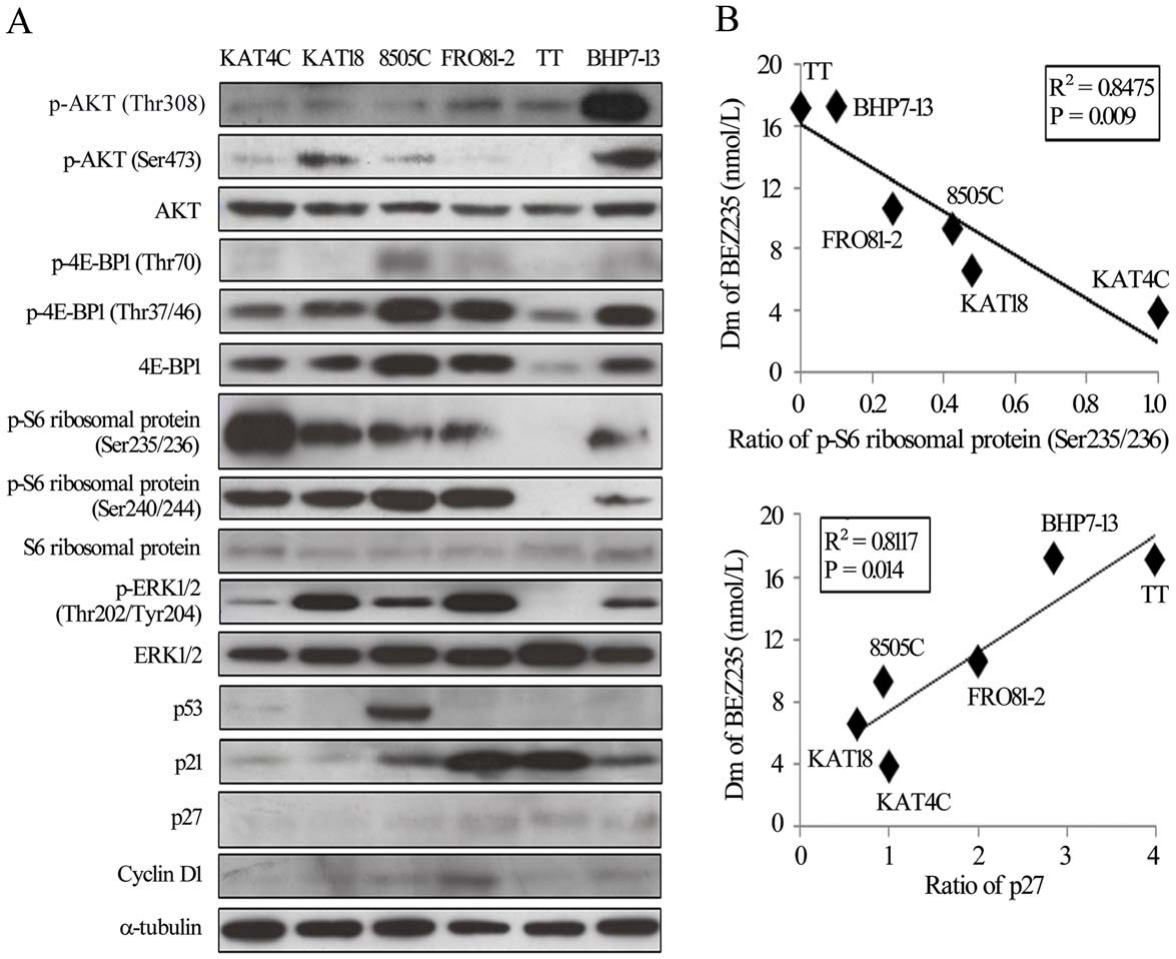
Susceptibility to BEZ235 correlates with baseline expression of p-S6 ribosomal protein (Ser235/236) and p27. A, the baseline levels of proteins associated with signaling transduction and cell cycle were evaluated by immunoblot. The sequence of proteins loaded was according to Dm. The expression of p-S6 ribosomal protein (Ser235/236) gradually decreased from KAT4C to BHP7-13, while p-27 showed the opposite order. The expression of the other proteins showed a random distribution. B, Band densities were imaged and quantified. Relationships between Dm and protein expression were analyzed using Pearson's correlation coefficients, and graphs were drawn using KAT4C. The expression of p-S6 ribosomal protein (Ser235/236) had a positive correlation, and p27 an inverse correlation, with sensitivity to BEZ235.

The Dm of BEZ235 spans a broad spectrum across 8 different cell lines, with an 11-fold difference between KAT4C and WRO82-1.To explore potential biomarkers that correlate with sensitivity of BEZ235, the baseline expression of proteins involved in the PI3K/mTOR and RAS/RAF/ERK pathways, and cell cycle-associated proteins were evaluated in 6 cell lines (Figure 4A). The sensitivity of the six cell lines to BEZ235 was ordered according to the Dm value.

The expression of p-S6 ribosomal protein (Ser235/236) gradually decreases across increasing Dm, while the level of p-27 gradually increases across increasing Dm. Statistical relationships were analyzed using Pearson's correlation coefficient (Figure 4B). The expression of p-S6 ribosomal protein (Ser235/236) had a significant correlation (R^2^ = 0.8475, P = 0.009) with BEZ235 sensitivity, while p27 had a significant inverse correlation (R^2^ = 0.8117, P = 0.014). A repeated immunoblot of p27 showed consistent results (Appendix S1). The expression of baseline p-AKT (Thr308), p-AKT (Ser473), p-4E-BP1(Thr70), p-4E-BP1 (Thr37/46), p-S6 ribosomal protein (Ser240/244), p-ERK1/2 (Thr202/Tyr204), p53, p21 and cyclin D1 failed to reveal any correlations with BEZ235 sensitivity (data not shown).

### BEZ235 therapy of murine flank tumors

**Figure 5:**
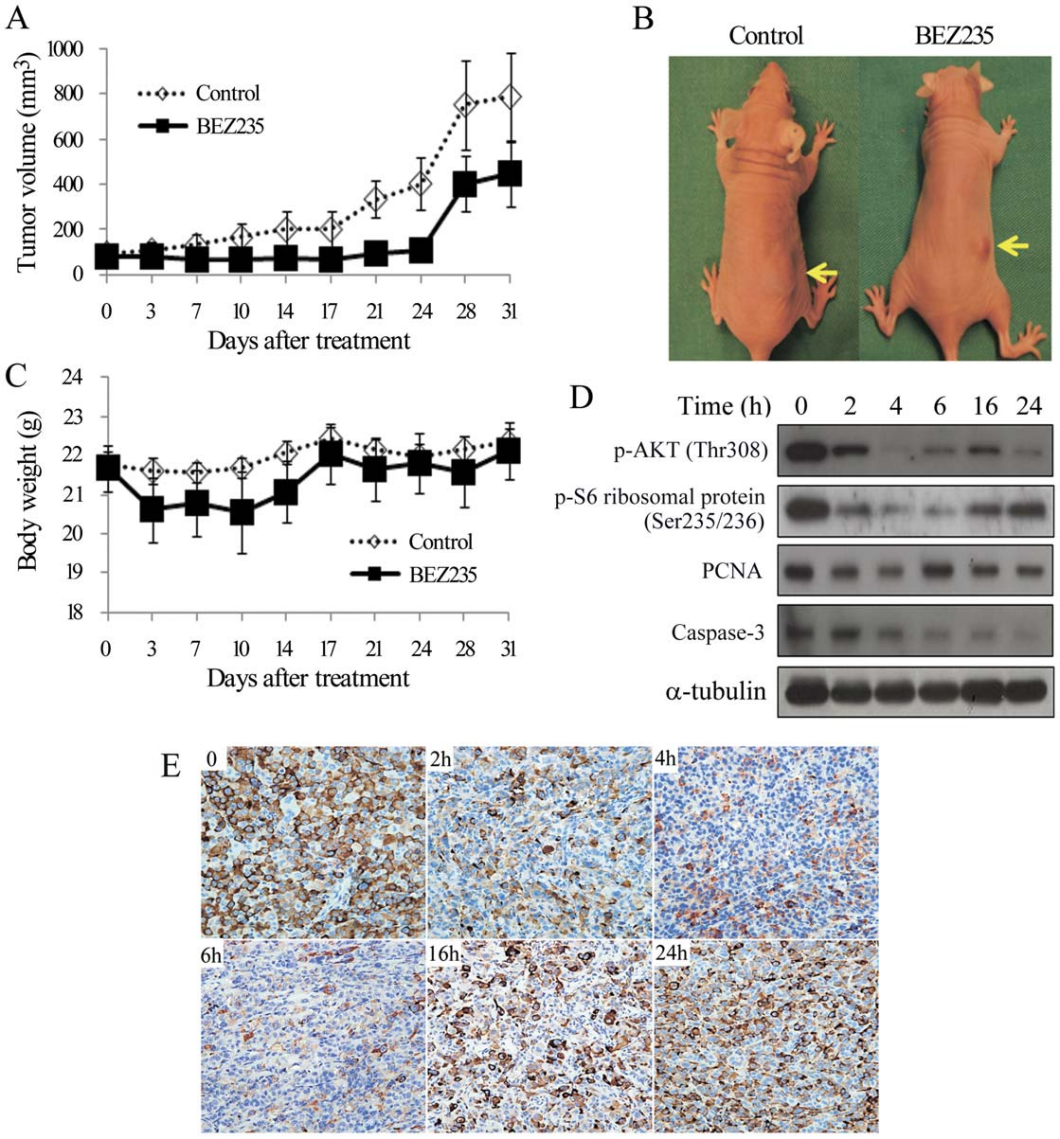
BEZ235 decreases p-AKT(Thr308), p-S6 ribosomal protein (Ser235/236), caspase-3, and retards the growth of ATC flank xenograft tumors without significant toxicity in nude mice. A, daily oral gavage of BEZ235 (50 mg/kg) represses 8505C tumor growth. The differences of tumor volume between BEZ235 and control group achieved statistical significance on days 21 and 24. Significance was lost on day 28, 4 days after treatment had been discontinued. B, representative mice with 8505C xenograft tumors (arrows) were photographed on last day of treatment (day 24). C, BEZ235 did not result in any weight loss, in treated and control mice (P>0.05 for both comparison). D, BEZ235 represses p-AKT (Thr308) and p-S6 ribosomal protein (Ser235/236) by 2 hours and caspase-3 by 4 hours in vivo. PCNA was slightly reduced at early period. E, expression of p-S6 ribosomal protein in tumor cells is detected by immunostaining at the indicated periods (magnification, ×200). These results were in consistent with Western blot results.

Athymic nude mice with flank xenografts of 8505C were used to study the therapeutic effects and safety of BEZ235 *in vivo*. Animals with established flank tumors of similar starting volumes were treated with oral BEZ235 (50 mg/kg) or vehicle daily for 25 days and followed until day 31 (Figure 5A). BEZ235 retarded tumor growth and the difference of tumor volumes between BEZ235 and control mice was statistically significant at day 21 (94.1±26.4 mm^3^ and 335.1±78.5 mm^3^, P = 0.009, t-test) and day 24 (109.9±29.6 mm^3^ and 403.1±112.8 mm^3^, P = 0.019, t-test). The difference of tumor volumes lost significance at day 28, 4 days after treatment had been discontinued (402.7±123.6 mm^3^ and 750.3±198.3 mm^3^, P = 0.15, t-test). Representative mice were photographed on the last day of treatment (Figure 5B).

BEZ235 did not significantly reduce body weight between the two groups during the study period (Figure 5C). Oral BEZ235 reduced phosphorylation of AKT and S6 ribosomal protein by 2 hours and achieved its greatest inhibitory effects at 4 hours with just 2% and 10% of pAKT and pS6 remaining, respectively. This inhibitory effect persisted for 24 hours in xenograft tumors (Figure 5D, Figure S5). PCNA was slightly and transiently repressed from 2 to 4 hours. However, significant degradation of caspase-3 occurred by 4 hours, with only 4% of caspase-3 remaining by 24 hours. Immunohistochemistry of p-S6 ribosomal protein (Ser235/236) in tumors treated with BEZ235 similarly demonstrated a reduction in staining, most markedly from 2 to 6 hours (Figure 5E).

### Interaction of BEZ235 and chemotherapy in ATC cells

**Figure 6:**
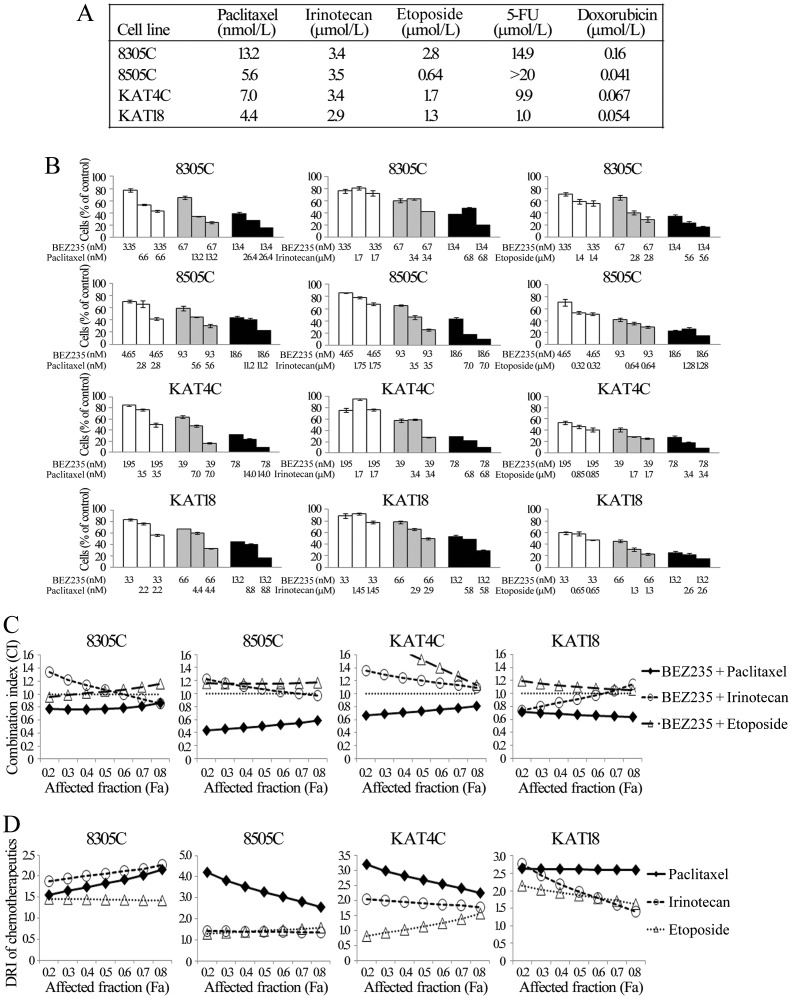
Combination therapy of BEZ235 with paclitaxel has synergistic effects against ATC. A, Dm was calculated using cytotoxicity data using CompuSyn software for each drug on each cell line at day 4. B, the cytotoxic effects of BEZ235 and chemotherapeutic agents (paclitaxel, irinotecan and etoposide) alone or in combination after a 4-day treatment in ATC were evaluated using LDH assays. The combination of BEZ235 and paclitaxel showed synergistic effects in all cell lines. The combination of BEZ235 and irinotecan had enhanced cytotoxic effects at higher doses, but revealed limited or no beneficial therapeutic effects at lower doses. The combination of BEZ235 plus etoposide displayed little additional cytotoxicity. C, the CI of BEZ235 and each chemotherapeutic agent was calculated using CompuSyn software. BEZ235 plus paclitaxel exhibited synergistic effects in all cell lines, with CI<0.8 at most conditions. BEZ235 plus irinotecan had synergistic to antagonistic effects in 8305C, 8505C and KAT18, and antagonistic effects in KAT4C. BEZ235 plus etoposide demonstrated mostly antagonistic effects in all cell lines. The horizontal dash lines at CI = 1 were drawn for discrimination of synergism (CI<1) and antagonism (CI>1). D, the typical ranges of DRI values for chemotherapeutics in combination with BEZ235. With the presence of BEZ235, paclitaxel mostly had the greatest DRIs in 8505C, KAT4C and KAT18, and also demonstrated favorable DRIs in 8305C.

We studied the combination of chemotherapeutic agents and BEZ235 against ATC. Five chemotherapeutic agents (paclitaxel, irinotecan, etoposide, 5-FU and doxorubicin) demonstrated cytotoxic effects in a dose- and time-dependent manner in four ATC cell lines (Figure S6). The data were used to calculate the median effect dose (Dm) on day 4 using CompuSyn software (Figure 6A).

These Dm of paclitaxel, irinotecan and etoposide are achievable in patients' serum, and are clinically relevant [32]–[34]. Although doxorubicin is used clinically for anaplastic thyroid cancer, the relatively high Dm in these cell lines implied that clinically relevant dosing is not achievable in patients serum and therefore was excluded from these studies. Interactions between BEZ235 and paclitaxel, irinotecan, and etoposide were evaluated (Figure 6B). The combination of BEZ235 and paclitaxel significantly improved cytotoxicity over single agent therapy in four cell lines. BEZ235 combined with irinotecan also enhanced therapeutic efficacy, particularly when more cells were affected. BEZ235 plus etoposide only slightly increased cytotoxicity.

Interactions between BEZ235 and each chemotherapeutic agent were determined by calculating the CI by Chou-Talalay equation (Figure 6C). Synergistic effects were identified for the combination of BEZ235 and paclitaxel in all ATC lines (CI, 8305C = 0.77–0.86, 8505C = 0.43–0.59, KAT4C = 0.66–0.81 and KAT18 = 0.64–0.71).

The combination of BEZ235 with irinotecan ranged from synergistic to antagonist in 8305C, 8505C and KAT18 (CI, 0.86–1.34, 0.97–1.23 and 0.74–1.14) and was antagonist in KAT4C (CI, 1.09–1.36). The combination of BEZ235 with etoposide was slightly synergistic to antagonist in 8305C (CI, 0.95–1.15) and antagonist in 8505C, KAT4C and KAT18 (CI, 1.16–1.17, 1.13–2.05 and 1.05–1.19, respectively).

These results demonstrate that BEZ235 and paclitaxel had the best combined effects in treating ATC. The calculated DRI of the chemotherapy agent means the fold of the drug dose that could be reduced in the presence of BEZ235 (Figure 6D). Among three chemotherapeutic agents, paclitaxel had the greatest DRIs in 8505C (2.55–4.2), KAT4C (2.25–3.19) and KAT18 (2.59–2.63), and also demonstrated favorable DRIs in 8305C (1.55–2.16).

## Discussion

BEZ235 effectively inhibited cell proliferation in eight thyroid cancer lines originating from four major histologic types. ATC were the most sensitive, followed by follicular undifferentiated, medullary and well-differentiated thyroid cancer cell lines. Cancer cells harboring a PI3K gain-of-function mutation or a *PTEN* deletion demonstrate higher PI3K/mTOR pathway activity and greater sensitivity to BEZ235 [22]. Our data suggest that ATC relies on PI3K/mTOR activity, and interruption of this pathway with BEZ235 impairs ATC growth more significantly than other thyroid cancer histologies. The relatively low median effect doses of BEZ235 in all of the thyroid cancer lines (<44 nmol/L) suggest that BEZ235 may have utility for treating a spectrum of thyroid malignancy. Refractory cancers that develop activation of PI3K/mTOR signaling in the process of tumor de-differentiation may be particularly attractive targets for therapy.

The failure of BEZ235 to repress p-AKT (Ser473) in 8505C and KAT4C may be related to a negative feedback inhibition. It has been previously shown that inhibition of mTORC1 leads to inactivation of S6 kinase 1, which may subsequently overwhelms the inhibitory effect of BEZ235 on mTORC2, activates mTORC2, and increases p-AKT (Ser473) in 8505C [35]. Prior reports also showed that lower doses of BEZ235 fail to inhibit p-AKT (Ser473) in some cell lines, and higher doses of BEZ235 may overcome the negative feedback of mTORC1/S6 kinase 1 feedback loop [18], [20]. Although p-AKT (Ser473) was activated in 8505C and KAT4C, BEZ235 had better inhibitory effects in these cell lines as compared to TT and BHP7-13, suggesting that other molecules affected by BEZ235 play a more important role in determining therapeutic outcome.

We found that the expression of p-S6 ribosomal protein (Ser235/236) and p27 correlate with the sensitivity of BEZ235 in thyroid cancer. S6 ribosomal protein is a downstream of S6 kinase 1, which is activated by mTORC1 [6]–[8]. Phosphorylation of S6 ribosomal protein increases translational control of protein synthesis and enhances cell growth. Cells with higher levels of p-S6 ribosomal protein (Ser235/236) are more susceptible to BEZ235, suggesting that the inhibition of mTORC1 and S6 ribosomal protein is the major therapeutic effect of BEZ235.

p-S6 ribosomal protein (Ser235/236) has also recently been recognized as a marker to predict therapeutic effect of an mTOR inhibitor in sarcoma [36]. In this study, thyroid cancer lines with higher expressions of p-S6 ribosomal protein (Ser235/236) also showed lower levels of p27. This finding suggests S6 ribosomal protein is a suppressor of p27 in thyroid cancer, and may explain why both p-S6 ribosomal protein (Ser235/236) and p27 were predictors of sensitivity to BEZ235. Interestingly, p27 was previously noted to have an inverse association with the activity of the PI3K/mTOR pathway in thyroid cancer cells, and repression of this pathway increases p27 [37].

4E-BP1 is another protein downstream of mTORC1. Inhibition of mTORC1 leads to dephosphorylation (activation) of 4E-BP1, enhancing the binding of 4E-BP1to eIF4E, and blocking protein translation and cell proliferation [7], [8]. Although BEZ235 affects both S6 ribosomal protein and 4E-BP1 efficiently, only p-S6 ribosomal protein (Ser235/236) expression predicts for sensitivity to BEZ235 in this study. In ovarian cancer, biomarkers predicting susceptibility of BEZ235 were reported [22], and the expression of p-4E-BP1 (Thr37/46) did correlate with the sensitivity of BEZ235.

In addition to inhibiting cell cycle progression, BEZ235 caused apoptosis in two of six cell lines. The inhibition of cell cycle progression is a known effect of BEZ235, even at lower doses (≤100 nmol/L). However, apoptosis appears in only some cancer cell lines and is more apparent at higher doses (100–1000 nmol/L) of BEZ235 [17]–[22]. BEZ235 efficiently inhibited mTORC1, a molecule controlling both cell cycle and apoptosis that might lead to cell cycle arrest and apoptosis in KAT4C and KAT18 [7]. Understanding the mechanisms through which BEZ235 contributes to apoptotic cell death will require further study.

ATC is by far the most aggressive of the four major histologic types of thyroid cancer. Chemotherapy has been applied to treat patients with ATC with response rates around 20 to 50%. Novel strategies to improve outcomes are needed. Among three combination therapy regimens, BEZ235 combined with paclitaxel had the best synergistic effect in four ATC cell lines. Cancer cells with activation of PI3K/mTOR signaling are more resistant to paclitaxel, and the co-administration of PI3K/mTOR inhibitors with paclitaxel improves therapeutic effects [38]–[40]. This finding is of clinical relevance since paclitaxel, a microtubule stabilizer, has shown to achieve a 53% response rate in patients with ATC in a phase II clinical trial [41]. The combination of BEZ235 with a microtubule depolymerizing drug vincristine also revealed promising effect in the treatment of sarcoma [20]. Our data showed that the combinational effects of BEZ235 with inhibitors of DNA topoisomerase type I (irinotecan) or type II (etoposide) in treating ATC were largely antagonistic. Similar antagonistic effects of inhibition both topoisomerase activity and PI3K/AKT pathway have been observed in ovarian cancer cells [42]. Cells in S phase are more susceptible to topoisomearse inhibitors. BEZ235-induced accumulation of cells at G0/G1 phase may therefore reduce the therapeutic advantage of concurrent therapy with topoisomerase inhibitors, explaining the unfavorable combination effects of BEZ235 with irinotecan and etoposide.

Daily treatment of BEZ235 significant retarded 8505C xenograft tumor growth during the therapeutic period. The inhibitory effect was less prominent after the discontinuation of therapy, suggesting that prolonged treatment may be necessary to maintain therapeutic efficacy. BEZ235 significantly degraded caspase-3 in 8505C xenograft tumors, indicating this compound may induce apoptosis *in vivo*. After discontinuation of BEZ235, the volume of 8505C xenograft tumors subsequently increased. This enlargement of tumor following cessation of therapy might be due to normalization of cell size and resumption of cell proliferation, as mTOR and its downstream proteins S6 Kinase 1 and 4E-BP1 play pivotal roles in this respect [43]. No significant weight loss or illness was observed during study period, suggesting that this therapy may have a promising safety profile.

A prior report demonstrated that the presence of genetic alterations of *PTEN*, *PIK3CA* and *AKT1* correlated well with sensitivity to an AKT inhibitor, but had weaker correlations with an mTOR inhibitor [44]. This discrepency suggests that mTOR activity does not depend solely on PI3K/AKT activity. The reported data of genetic alterations in 8 thyroid cancer cell lines was summarized (Appendix S2). Limited genetic changes on RAS/RAF/ERK and PI3K/mTOR pathways were identified. The available data do not show any correlation between genetic aberrations of PI3K/mTOR pathways and sensitivity of BEZ235, as no mutation of this pathway in these cell lines reported. Mutations of p53 and BRAF also seem to not be correlated with sensitivity of BEZ235, because both sensitive (8305C) and less sensitive (WRO82-1) cell lines harbor the mutations. Exploration of genetic changes of mTOR, the major target of BEZ235, might someday provide information that may predict therapeutic efficacy.

Inhibition of mTORC1 may activate the MAPK pathway through a PI3K-dependent feedback loop [45], which explains why BEZ235 was seen here to activate ERK1/2 in thyroid cancer cells. Combining BEZ235 with an inhibitor targeting MAPK pathway may be a potential approach to enhance therapeutic efficacy. Recently, the combination of a pan-RAF inhibitor and BEZ235 was shown to induce cell cycle arrest at G0/G1 phase, and had beneficial combination effects in treating thyroid cancer [46]. Similarly, in combination of mTOR and MEK inhibitors also demonstrated therapeutic advantage in treating thyroid cancer, providing further evidence that targeting both PI3K/mTOR and MAPK pathways is a potential therapeutic strategy for this disease [47], [48].

## Materials and Methods

### Cell lines

Eight cell lines were obtained, including a papillary (BHP7-13), a follicular (WRO82-1), a follicular undifferentiated (FRO81-2), four anaplastic (8505C, 8305C, KAT4C, KAT18) and a medullary (TT) human thyroid cancer [23]–[25]. All cell lines except KAT4C were authenticated using DNA (short tandem repeats) profiling and stored in liquid nitrogen [26]. BHP7-13, WRO82-1, FR081-2, KAT4C and KAT18 were maintained in RPMI 1640 with sodium bicarbonate (2.0 g/L). 8505C and 8305C were maintained in MEM with sodium pyruvate (1 mmol/L) and sodium bicarbonate (2.2 g/L). TT was maintained in F12K. All media contained 10% FCS, 100,000 units/L penicillin and 100 mg/L streptomycin. All cells were maintained in a 5% CO2 humidified incubator at 37°C.

### Pharmacologic agents

BEZ235 is a dual PI3K/mTOR inhibitor described previously [17], and was a generous gift from Novartis Pharma AG (Basel, Switzerland). BEZ235 (10 mmol/L) was dissolved in DMSO (Sigma) and stored at −30°C until *in vitro* experiments. For *in vivo* studies, BEZ235 was diluted with 1-Methyl-2-pyrrolidone (NMP; Sigma) and poly(ethylene glycol) (average M_n_ 285–315; Sigma) (1∶9 v/v) to a final concentration of 21.5 mg/ml. Paclitaxel, irinotecan, etoposide, 5-Fluorouracil (5-FU) and doxorubicin were obtained from Sigma Chemical Co. Paclitaxel (320 µmol/L), irinotecan (16 mmol/L), etoposide (5 mmol/L) and 5-FU (40 mmol/L) were dissolved in DMSO and doxorubicin (5 mmol/L) was dissolved in PBS and stored at −30°C until use.

### Antibodies

Antibodies targeting p-AKT (Thr308), p-AKT (Ser473), AKT, p-4E-BP1 (Thr70), p-4E-BP1 (Thr37/46), 4E-BP1, p-S6 ribosomal proteins (Ser235/236), p-S6 ribosomal protein (Ser240/244), S6 ribosomal protein, p-ERK1/2 (Thr202/Tyr204), ERK1/2, p53, p21, p27, cyclin D1, caspase-3 and proliferating cell nuclear antigen (PCNA) were from Cell Signaling Technology. α-tubulin antibody was from Sigma.

### Cytotoxicity assays

Cells were plated at 2×10^4^ cells per well in 24-well plates in 1 mL media. After overnight incubation, the agents or vehicle were added at the indicated concentration. Six serial 1∶1 dilutions were tested starting at the following doses: BEZ235 at 100 nmol/L, paclitaxel at 68 nmol/L (8305C, 8505C and KAT4C) and 20 nmol/L (KAT18), irinotecan at 8 µmol/L, etoposide at 10 µmol/L, 5-FU at 20 µmol/L and doxorubicin at 1 µmol/L over a 4-day treatment course. Cytotoxicity was determined daily for BEZ235 group and on day 3 and 4 for the chemotherapy groups. Cells were washed with PBS and lysed with Triton X-100 (1.35%, Sigma) to release intracellular lactate dehydrogenase (LDH), which was quantified with a Cytotox 96 kit (Progmega) at 490 nM by spectrophotometry (BT-MQX200R, Bio-Tek Instruments). Each experiment was performed in triplicate and results are shown as the percentage of surviving cells determined by comparing the LDH of each sample relative to control samples which are considered 100% viable. Median effect doses (Dm) on day 4 were calculated for each cell line and drug using CompuSyn software [27].

For combination therapy experiments, ATC cells were treated with BEZ235 and a chemotherapeutic drug (paclitaxel, irinotecan or etoposide) at a fixed dose ratio. Cells were incubated with vehicle, BEZ235, chemotherapeutic agent, or both simultaneously for a 4-day course and cytotoxicity was measured. Five serial 1∶1 dilutions were examined at the following starting doses for 8305C, 8505C, KAT4C and KAT18: BEZ235 at 26.8, 37.2, 15.6 and 26.4 nmol/L, paclitaxel at 52.8, 22.4, 28 and 17.6 nmol/L, irinotecan at 13.6, 14, 13.6 and 11.6 µmol/L, etoposide at 11.2, 2.56, 6.8 and 5.2 µmol/L, respectively. The doses chosen were based on the Dm determined previously.

### Western blots

Cells were plated at 8×10^5^ cells in 100-mm Petri dishes in 8 mL media overnight and treated with BEZ235 or vehicle for indicated periods. Cells were plated overnight for baseline expression of untreated cells. Cell pellets were dissolved in radio-immunoprecipitation assay buffer and protease inhibitor cocktail, vortexed and clarified by centrifugation. Total protein (20–50 µg) was electrophoresed on 10–12% Tris-HCl gels, transferred to polyvinylidene difluoride membranes, blocked, and exposed to primary antibodies followed by a secondary antibody conjugated to horseradish peroxidase. Signals were developed using an enhanced chemiluminescence kit (Bionovas Biotechnology).

Band density was imaged and quantified using Molecular Imager VersaDoc MP 4000 system (Bio-Rad). The ratios of p-AKT, p-4E-BP1, p-S6 ribosomal protein and p-ERK1/2 to the correlated total protein, and the ratios of p53, p21, p27 and cyclin D1 to α-tubulin in each cell line were calculated. Relative expression was calculated using untreated cells and KAT4C baseline values as a reference, or as indicated otherwise.

### Cell cycle and apoptosis assessment

Cells were plated at 1×10^5^ cells per well in 6-well plates in 2 mL media overnight. BEZ235 or vehicle were added at indicated doses for 24 hours (KAT4C, 8505C, BHP7-13 and WRO82-1) or 72 hours (TT), adherent cells were trypsinized, washed with PBS, fixed with cold 70% ethanol and incubated with RNase A (100 µg/mL; Sigma) and propidium iodide (5 µg/mL; Sigma) at 37°C for 15 min. Cell cycle distribution was assessed by DNA content detected by flow cytometry (BD FACScalibur Flow Cytometer, BD Biosciences).

Cells were plated at 2×10^4^ cells per well in 6-well plates in 2 mL media overnight and treated with BEZ235 for 96 hours. Floating cells and trypsinized adherent cells were collected and samples were prepared as described above. Apoptotic sub-G1 cells were detected by DNA content by flow cytometry. Each condition was performed in triplicate.

### Flank xenograft tumor therapy

Eight-week-old athymic female nude mice (National Laboratory Animal Center, Taiwan) were anesthetized with intraperitoneal injection of ketamine hydrochloride (90 mg/kg; Nang Kuang Pharmaceutical Co.) and xylazine hydrochloride (9 mg/kg; Bayer) before implantation of thyroid cancer cells. 8505C flank tumors were established by injecting 1×10^6^ cells in 100 µL PBS into the subcutaneous flanks of nude mice. When tumors reached 6 mm in mean diameter, mice (*n* = 7–8 per group) were treated with BEZ235 (50 mg/kg) or placebo by oral gavage daily for 25 days. Tumor dimensions were serially measured with electronic calipers twice a week, and the volumes were calculated by the formula a^2^×b×0.4, where a represents the smallest diameter and b is the perpendicular diameter. The body weight of each animal was followed as a marker of toxicity.

Tumor levels of p-AKT, p-S6 ribosomal protein, PCNA and caspase-3 were evaluated in mice treated with a single dose of BEZ235 (50 mg/kg). At indicated periods, carbon dioxide was used for euthanasia, tumors were then harvested, mixed with protein extraction buffer (GE Healthcare), homogenized and sonicated on ice. After centrifugation, clarified supernatants were aliquoted and stored at −80°C until western blot was performed. Tumors were also fixed in 10% formalin and paraffin embedded. Sections (5 µm) were incubated with rabbit p-S6ribosomal protein antibody (1∶100) at room temperature for 30 minutes, followed by poly-horseradish peroxidase anti-rabbit IgG reagent, and diaminobenzidine utilized to visualize the complex. Sections were counterstained with hematoxylin. This study was performed in strict accordance with the recommendations in the Guide for the Care and Use of Laboratory Animals of Chang Gung Memorial Hospital and the protocol was approved by the Committee of Laboratory Animal Center at Chang Gung Memorial Hospital, Linkou (Permit Number: 2008112401).

### Quantitative analysis of drug interactions and statistical analyses

Interactions between BEZ235 and the chemotherapeutic drugs for each cell line were determined by calculating the combination index (CI) of Chou-Talalay equation where CI<1 is synergism,  = 1 is addictive, and >1 is antagonism [28], [29]. The dose-effect analysis was produced using the computer software, CompuSyn [27], [30], following the dose and effect data entries. The dose reduction index (DRI) was also determined. DRI represents the fold dose-reduction permitted by the combination, for a given effect level, when compared with each drug alone [30].

Comparisons were performed when appropriate using two-sided Student's *t* test, and correlations using Pearson's coefficients (Excel, Microsoft). Results were expressed as the mean ± SE.

In conclusion, BEZ235 effectively inhibits the proliferation of four different histologic types of thyroid cancer. The therapeutic effect and safety profiles are favorable in nude mice bearing 8505C xenograft tumors. Importantly, BEZ235 synergistically enhances the therapeutic effect of paclitaxel in treating ATC. These data support future clinical trials investigating the utility of BEZ235 as an agent to treat patients with refractory thyroid cancer.

## Supporting Information

Appendix S1 Basal expression of p27 in thyroid cancer cell lines.
(TIF)

Appendix S2 Reported data of genetic alterations in thyroid cancer cell lines and Dm of BEZ235.
(TIF)

Figure S1 BEZ235 consistently inhibits mTORC1 signaling and activates p-ERK1/2. A, p-AKT (Thr308) was transient reduced by 2 hours in TT and BHP7-13 and increased by 4 hours in 8505C and KAT4C. B, p-AKT (Ser473) was reduced by 2 and 8 hours in TT and BHP7-13 and increased by 2 hours in 8505C and KAT4C. C, p-4E-BP1 (Thr70) was consistently reduced by 2 hours in 3 cell lines. D, p-4E-BP1 (Thr37/46) was reduced by 2 hours. The extent of repression is statistically significant from 2 to 24 hours in BHP7-13 compared with baseline level. E, p-S6 ribosomal protein (Ser235/236) was decreased by 2 hours. The inhibition was significant in 8505C and TT from 4 to 24 hours compared with basal level. F, p-ERK1/2 (Thr202/Tyr204) was activated by 2 to 4 hours in four cell lines. * denoted P<0.02.
(TIF)

Figure S2 BEZ235 inhibits p-S6 ribosomal protein (Ser235/236) and activates p-ERK1/2 and p27 in KAT4C. A, p-AKT (Thr308), p-AKT (Ser473) and p-ERK1/2 (Thr202/Tyr204) was increased by 4 hours and persisted for more than 24 hours. p-S6 ribosomal protein (Ser235/236) was reduced from 8 hours through over 24 hours. B, p27 was increased by 24 hours. p53, p21 and cyclin D1 were increased by 4 to 24 hours.
(TIF)

Figure S3 BEZ235 activates p27 expression in thyroid cancer cell lines. A, p53was decreased by 2 to 4 hours in 8505C and TT and increased in KAT4C by 8 hours. B, p21 was repressed in TT and BHP7-13 and increased in KAT4C by 8 hour. C, p27 was increased in all cell lines. The elevation of p27 achieved statistical significance at 8 hour in TT. There was no detectable signal in basal 8505C cells, therefore the reference data was at 4 h. D, Cyclin D1 was gradually decreased in 8505C and BHP7-13 and increased at certain time points in KAT4C and TT. There was no detectable signal in untreated KAT4C, therefore the reference data was 8 h. * denoted P = 0.019 (t-test).
(TIF)

Figure S4 BEZ235 degrades caspase-3 *in vitro*. Quantification of immunoblot showed caspase-3 was decreased in KAT4C and KAT18 at 72 hours at doses ranged from 6.25 to 100 nmol/L.
(TIF)

Figure S5 BEZ235 represses the expression of p-AKT, p-S6 ribosomal protein and capase-3 in vivo. The effects of BEZ235 on p-AKT, p-S6 ribosomal protein, PCNA and capase-3 in vivo. Quantification of immunoblot showed BEZ235 greatly repressed p-AKT (Thr308), p-S6 ribosomal protein (Ser235/236) and caspase-3 by 2 to 4 hours with durable effects. PCNA was slightly reduced at an early time point.
(TIF)

Figure S6 Five chemotherapeutic agents induce dose and time dependent cytotoxicity in 4 anaplastic thyroid cancer cell lines. Dose-response curves were obtained on day 3 and 4 from cells treated with serial dilutions of chemotherapeutic agents (paclitaxel, irinotecan, etoposide, 5-FU, doxorubicin) for a 4-day course on ATC cell lines using LDH assays. All drugs demonstrated dose and time dependent cytotoxicity in four cell lines.
(TIF)

## References

[1] Davies L, Welch HG (2006) Increasing incidence of thyroid cancer in the United States, 1973–2002. JAMA 295: 2164–2167. 16684987 10.1001/jama.295.18.2164

[2] Chen AY, Jemal A, Ward EM (2009) Increasing incidence of differentiated thyroid cancer in the United States, 1988–2005. Cancer 115: 3801–3807. 19598221 10.1002/cncr.24416

[3] Ricarte-Filho JC, Ryder M, Chitale DA, Rivera M, Heguy A, et al (2009) Mutational profile of advanced primary and metastatic radioactive iodine-refractory thyroid cancers reveals distinct pathogenetic roles for BRAF, PIK3CA, and AKT1. Cancer Res 69: 4885–4893. 19487299 10.1158/0008-5472.CAN-09-0727 PMC2690720

[4] Nikiforov YE, Nikiforova MN (2011) Molecular genetics and diagnosis of thyroid cancer. Nat Rev Endocrinol 7: 569–580. 21878896 10.1038/nrendo.2011.142

[5] Sippel RS, Kunnimalaiyaan M, Chen H (2008) Current management of medullary thyroid cancer. Oncologist 13: 539–547. 18515739 10.1634/theoncologist.2007-0239

[6] Engelman JA (2009) Targeting PI3K signalling in cancer: opportunities, challenges and limitations. Nat Rev Cancer 9: 550–562. 19629070 10.1038/nrc2664

[7] Faivre S, Kroemer G, Raymond E (2006) Current development of mTOR inhibitors as anticancer agents. Nat Rev Drug Discov 5: 671–688. 16883305 10.1038/nrd2062

[8] Hennessy BT, Smith DL, Ram PT, Lu Y, Mills GB (2005) Exploiting the PI3K/AKT pathway for cancer drug discovery. Nat Rev Drug Discov 4: 988–1004. 16341064 10.1038/nrd1902

[9] Wu G, Mambo E, Guo Z, Hu S, Huang X, et al (2005) Uncommon mutation, but common amplifications, of the PIK3CA gene in thyroid tumors. J Clin Endocrinol Metab 90: 4688–4693. 15928251 10.1210/jc.2004-2281

[10] Hou P, Liu D, Shan Y, Hu S, Studeman K, et al (2007) Genetic alterations and their relationship in the phosphatidylinositol 3-kinase/Akt pathway in thyroid cance. Clin Cancer Res 13: 1161–1170. 17317825 10.1158/1078-0432.CCR-06-1125

[11] Santarpia L, El-Naggar AK, Cote GJ, Myers JN, Sherman SI (2008) Phosphatidylinositol 3-kinase/akt and ras/raf-mitogen-activated protein kinase pathway mutations in anaplastic thyroid cancer. J Clin Endocrinol Metab 93: 278–284. 17989125 10.1210/jc.2007-1076

[12] Liu Z, Hou P, Ji M, Guan H, Studeman K, et al (2008) Highly prevalent genetic alterations in receptor tyrosine kinases and phosphatidylinositol 3-kinase/akt and mitogen-activated protein kinase pathways in anaplastic and follicular thyroid cancers. J Clin Endocrinol Metab 93: 3106–3116. 18492751 10.1210/jc.2008-0273

[13] Abubaker J, Jehan Z, Bavi P, Sultana M, Al-Harbi S, et al (2008) Clinicopathological analysis of papillary thyroid cancer with PIK3CA alterations in a Middle Eastern population. J Clin Endocrinol Metab 93: 611–618. 18000091 10.1210/jc.2007-1717

[14] Wang Y, Hou P, Yu H, Wang W, Ji M, et al (2007) High prevalence and mutual exclusivity of genetic alterations in the phosphatidylinositol-3-kinase/akt pathway in thyroid tumors. J Clin Endocrinol Metab 92: 2387–2390. 17426084 10.1210/jc.2006-2019

[15] Carlomagno F, Santoro M (2011) Thyroid cancer in 2010: a roadmap for targeted therapies. Nat Rev Endocrinol 7: 65–67. 21263433 10.1038/nrendo.2010.232

[16] Rapa I, Saggiorato E, Giachino D, Palestini N, Orlandi F, et al (2011) Mammalian target of rapamycin pathway activation is associated to RET mutation status in medullary thyroid carcinoma. J Clin Endocrinol Metab 96: 2146–2153. 21543427 10.1210/jc.2010-2655

[17] Maira SM, Stauffer F, Brueggen J, Furet P, Schnell C, et al (2008) Identification and characterization of NVP-BEZ235, a new orally available dual phosphatidylinositol 3-kinase/mammalian target of rapamycin inhibitor with potent in vivo antitumor activity. Mol Cancer Ther 7: 1851–1863. 18606717 10.1158/1535-7163.MCT-08-0017

[18] Serra V, Markman B, Scaltriti M, Eichhorn PJ, Valero V, et al (2008) NVP-BEZ235, a dual PI3K/mTOR inhibitor, prevents PI3K signaling and inhibits the growth of cancer cells with activating PI3K mutations. Cancer Res 68: 8022–8030. 18829560 10.1158/0008-5472.CAN-08-1385

[19] Marone R, Erhart D, Mertz AC, Bohnacker T, Schnell C, et al (2009) Targeting melanoma with dual phosphoinositide 3-kinase/mammalian target of rapamycin inhibitors. Mol Cancer Res 7: 601–613. 19372588 10.1158/1541-7786.MCR-08-0366

[20] Manara MC, Nicoletti G, Zambelli D, Ventura S, Guerzoni C, et al (2010) NVP-BEZ235 as a new therapeutic option for sarcomas. Clin Cancer Res 16: 530–540. 20068094 10.1158/1078-0432.CCR-09-0816

[21] Chapuis N, Tamburini J, Green AS, Vignon C, Bardet V, et al (2010) Dual inhibition of PI3K and mTORC1/2 signaling by NVP-BEZ235 as a new therapeutic strategy for acute myeloid leukemia. Clin Cancer Res 16: 5424–5435. 20884625 10.1158/1078-0432.CCR-10-1102

[22] Santiskulvong C, Konecny GE, Fekete M, Chen KY, Karam A, et al (2011) Dual targeting of phosphoinositide 3-kinase and mammalian target of rapamycin using NVP-BEZ235 as a novel therapeutic approach in human ovarian carcinoma. Clin Cancer Res 17: 2373–2384. 21372221 10.1158/1078-0432.CCR-10-2289 PMC3078990

[23] Ohta K, Pang XP, Berg L, Hershman JM (1997) Growth inhibition of new human thyroid carcinoma cell lines by activation of adenylate cyclase through the beta-adrenergic receptor. J Clin Endocrinol Metab 82: 2633–2638. 9253346 10.1210/jcem.82.8.4136

[24] Huang YY, Yu Z, Lin SF, Li S, Fong Y, et al (2007) Nectin-1 is a marker of thyroid cancer sensitivity to herpes oncolytic therapy. J Clin Endocrinol Metab 92: 1965–1970. 17327376 10.1210/jc.2007-0040

[25] Lin SF, Yu Z, Riedl C, Woo Y, Zhang Q, et al (2007) Treatment of anaplastic thyroid carcinoma in vitro with a mutant vaccinia virus. Surgery 142: 976–983 discussion 976–983. 18063085 10.1016/j.surg.2007.09.017

[26] Schweppe RE, Klopper JP, Korch C, Pugazhenthi U, Benezra M, et al (2008) Deoxyribonucleic acid profiling analysis of 40 human thyroid cancer cell lines reveals cross-contamination resulting in cell line redundancy and misidentification. J Clin Endocrinol Metab 93: 4331–4341. 18713817 10.1210/jc.2008-1102 PMC2582569

[27] Chou TC, Martin N (2005) CompuSyn for Drug Combinations: PC Software and User's Guide: A Computer Program for Quantitation of Synergism and Antagonism in Drug Combinations, and the Determination of IC50 and ED50 and LD50 Values. Paramus: ComboSyn.

[28] Chou TC, Talalay P (1984) Quantitative analysis of dose-effect relationships: the combined effects of multiple drugs or enzyme inhibitors. Adv Enzyme Regul 22: 27–55. 6382953 10.1016/0065-2571(84)90007-4

[29] Chou TC (2006) Theoretical basis, experimental design, and computerized simulation of synergism and antagonism in drug combination studies. Pharmacol Rev 58: 621–681. 16968952 10.1124/pr.58.3.10

[30] Chou TC (2010) Drug combination studies and their synergy quantification using the Chou-Talalay method. Cancer Res 70: 440–446. 20068163 10.1158/0008-5472.CAN-09-1947

[31] Lapenna S, Giordano A (2009) Cell cycle kinases as therapeutic targets for cancer. Nat Rev Drug Discov 8: 547–566. 19568282 10.1038/nrd2907

[32] Hughes AN, Griffin MJ, Newell DR, Calvert AH, Johnston A, et al (2000) Clinical pharmacokinetic and in vitro combination studies of nolatrexeddihydrochloride (AG337, Thymitaq) and paclitaxel. Br J Cancer 82: 1519–1527. 10789718 10.1054/bjoc.2000.1172 PMC2363406

[33] Rothenberg ML, Kuhn JG, Schaaf LJ, Rodriguez GI, Eckhardt SG, et al (2001) Phase I dose-finding and pharmacokinetic trial of irinotecan (CPT-11) administered every two weeks. Ann Oncol 12: 1631–1641. 11822765 10.1023/a:1013157727506

[34] Sprinz E, Caldas AP, Mans DR, Cancela A, DiLeone L, et al (2001) Fractionated doses of oral etoposide in the treatment of patients with aids-related kaposi sarcoma: a clinical and pharmacologic study to improve therapeutic index. Am J Clin Oncol 24: 177–184. 11319295 10.1097/00000421-200104000-00016

[35] Foster KG, Fingar DC (2010) Mammalian target of rapamycin (mTOR): conducting the cellular signaling symphony. J Biol Chem 285: 14071–14077. 20231296 10.1074/jbc.R109.094003 PMC2863215

[36] Iwenofu OH, Lackman RD, Staddon AP, Goodwin DG, Haupt HM, et al (2008) Phospho-S6 ribosomal protein: a potential new predictive sarcoma marker for targeted mTOR therapy. Mod Pathol 21: 231–237. 18157089 10.1038/modpathol.3800995

[37] Motti ML, Califano D, Troncone G, De Marco C, Migliaccio I, et al (2005) Complex regulation of the cyclin-dependent kinase inhibitor p27kip1 in thyroid cancer cells by the PI3K/AKT pathway: regulation of p27kip1 expression and localization. Am J Pathol 166: 737–749. 15743786 10.1016/S0002-9440(10)62295-X PMC1602368

[38] Isakoff SJ, Engelman JA, Irie HY, Luo J, Brachmann SM, et al (2005) Breast cancer-associated PIK3CA mutations are oncogenic in mammary epithelial cells. Cancer Res 65: 10992–11000. 16322248 10.1158/0008-5472.CAN-05-2612

[39] Kim SH, Juhnn YS, Song YS (2007) Akt involvement in paclitaxel chemoresistance of human ovarian cancer cells. Ann N Y Acad Sci 1095: 82–89. 17404021 10.1196/annals.1397.012

[40] Shafer A, Zhou C, Gehrig PA, Boggess JF, Bae-Jump VL (2010) Rapamycin potentiates the effects of paclitaxel in endometrial cancer cells through inhibition of cell proliferation and induction of apoptosis. Int J Cancer 126: 1144–1154. 19688827 10.1002/ijc.24837 PMC2818608

[41] Ain KB, Egorin MJ, DeSimone PA (2000) Treatment of anaplastic thyroid carcinoma with paclitaxel: phase 2 trial using ninety-six-hour infusion. Collaborative Anaplastic Thyroid Cancer Health Intervention Trials (CATCHIT) Group. Thyroid 10: 587–594. 10958311 10.1089/thy.2000.10.587

[42] Fekete M, Santiskulvong C, Eng C, Dorigo O (2012) Effect of PI3K/Akt pathway inhibition-mediated G1 arrest on chemosensitization in ovarian cancer cells. Anticancer Res 32: 445–452. 22287731

[43] Fingar DC, Salama S, Tsou C, Harlow E, Blenis J (2002) Mammalian cell size is controlled by mTOR and its downstream targets S6K1 and 4EBP1/eIF4E. Genes Dev 16: 1472–1487. 12080086 10.1101/gad.995802 PMC186342

[44] Liu Z, Wu G, Xing M (2009) Genetic alterations in the phosphoinositide 3-kinase/Akt signaling pathway confer sensitivity of thyroid cancer cells to therapeutic targeting of Akt and mammalian target of rapamycin. Cancer Res 69: 7311–7319. 19706758 10.1158/0008-5472.CAN-09-1077 PMC2756336

[45] Carracedo A, Ma L, Teruya-Feldstein J, Rojo F, Salmena L, et al (2008) Inhibition of mTORC1 leads to MAPK pathway activation through a PI3K-dependent feedback loop in human cancer. J Clin Invest 118: 3065–3074. 18725988 10.1172/JCI34739 PMC2518073

[46] Jin N, Jiang T, Rosen DM, Nelkin BD, Ball DW (2011) Synergistic action of a RAF inhibitor and a dual PI-3 Kinase/mTOR inhibitor in thyroid cancer. Clin Cancer Res 17: 6482–6489. 21831957 10.1158/1078-0432.CCR-11-0933 PMC4828042

[47] Liu D, Xing J, Trink B, Xing M (2010) BRAF mutation-selective inhibition of thyroid cancer cells by the novel MEK inhibitor RDEA119 and genetic-potentiated synergism with the mTOR inhibitor temsirolimus. Int J Cancer 127: 2965–2973. 21351275 10.1002/ijc.25304 PMC2916062

[48] Jin N, Jiang T, Rosen DM, Nelkin BD, Ball DW (2009) Dual inhibition of mitogen-activated protein kinase kinase and mammalian target of rapamycin in differentiated and anaplastic thyroid cancer. J Clin Endocrinol Metab 94: 4107–4112. 19723757 10.1210/jc.2009-0662 PMC2758734

