# Supplementary figures and images for: Utility of a PI3K/mTOR Inhibitor (NVP-BEZ235) for Thyroid Cancer Therapy
Source: PLoS One. 2012 Oct 15;7(10):e46726. doi: 10.1371/journal.pone.0046726 (PMC3471922; doi:10.1371/journal.pone.0046726)

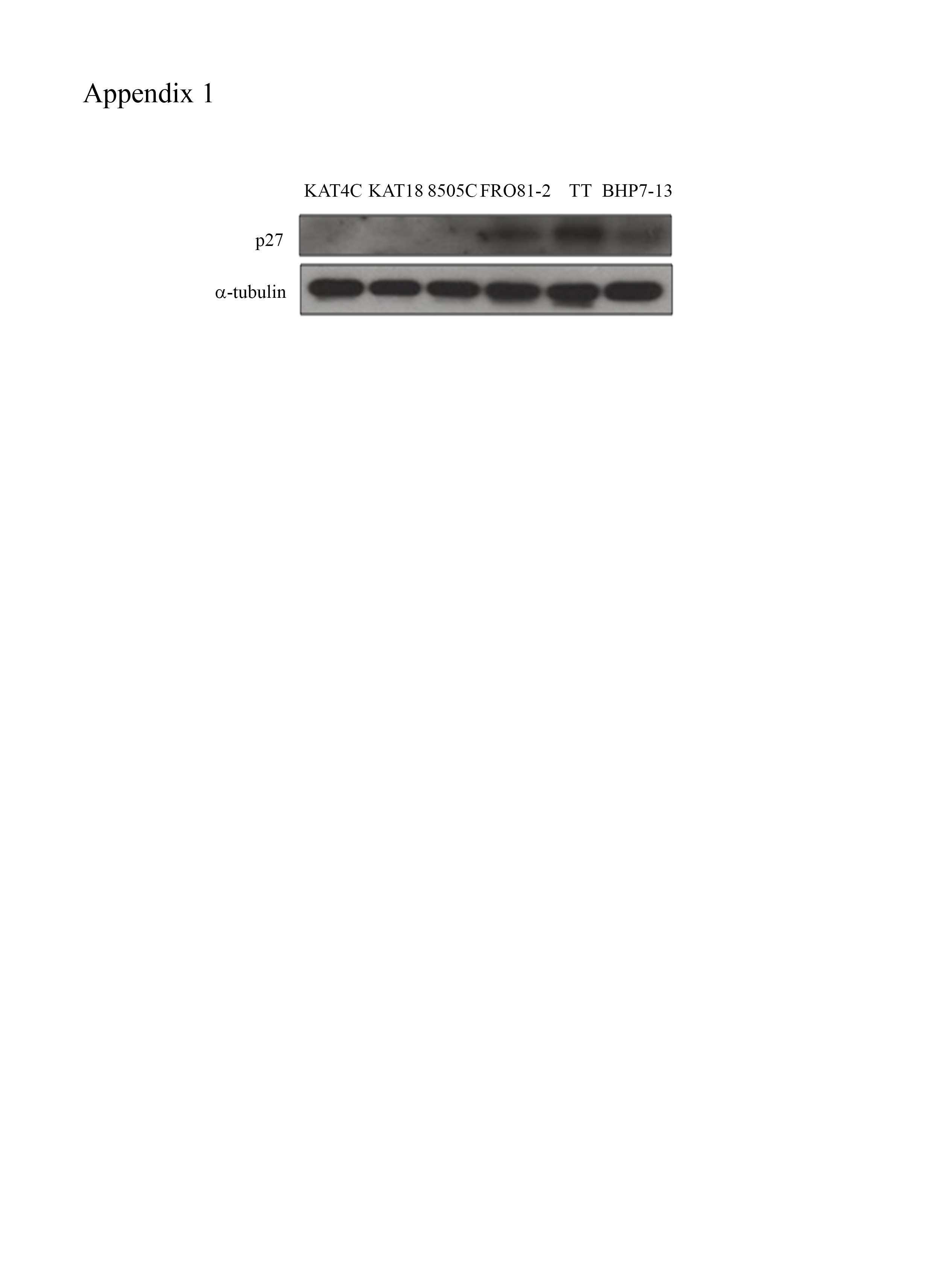

Supplement: Appendix S1 — Basal expression of p27 in thyroid cancer cell lines. (TIF) [file pone.0046726.s001.tif]

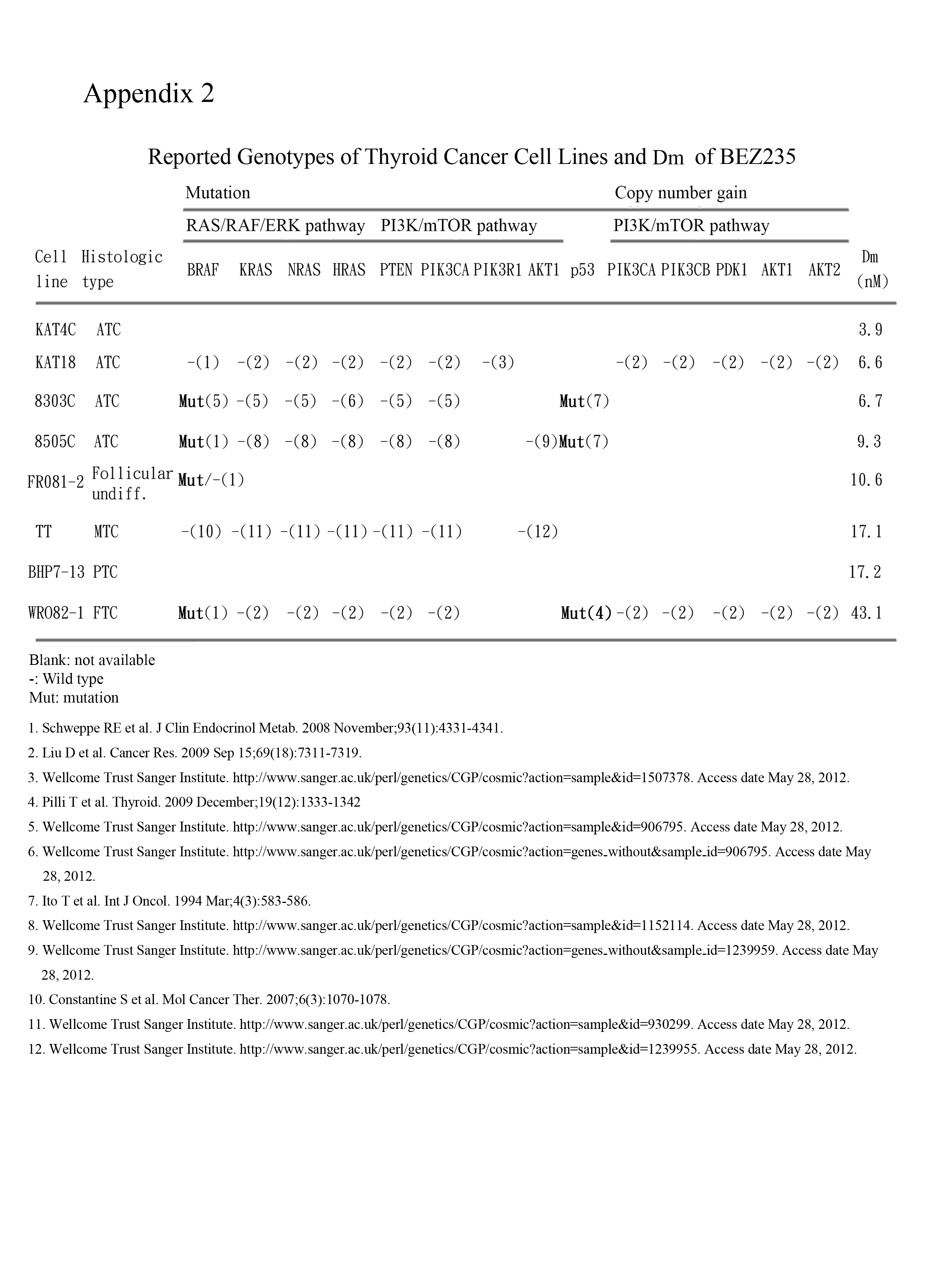

Supplement: Appendix S2 — Reported data of genetic alterations in thyroid cancer cell lines and Dm of BEZ235. (TIF) [file pone.0046726.s002.tif]

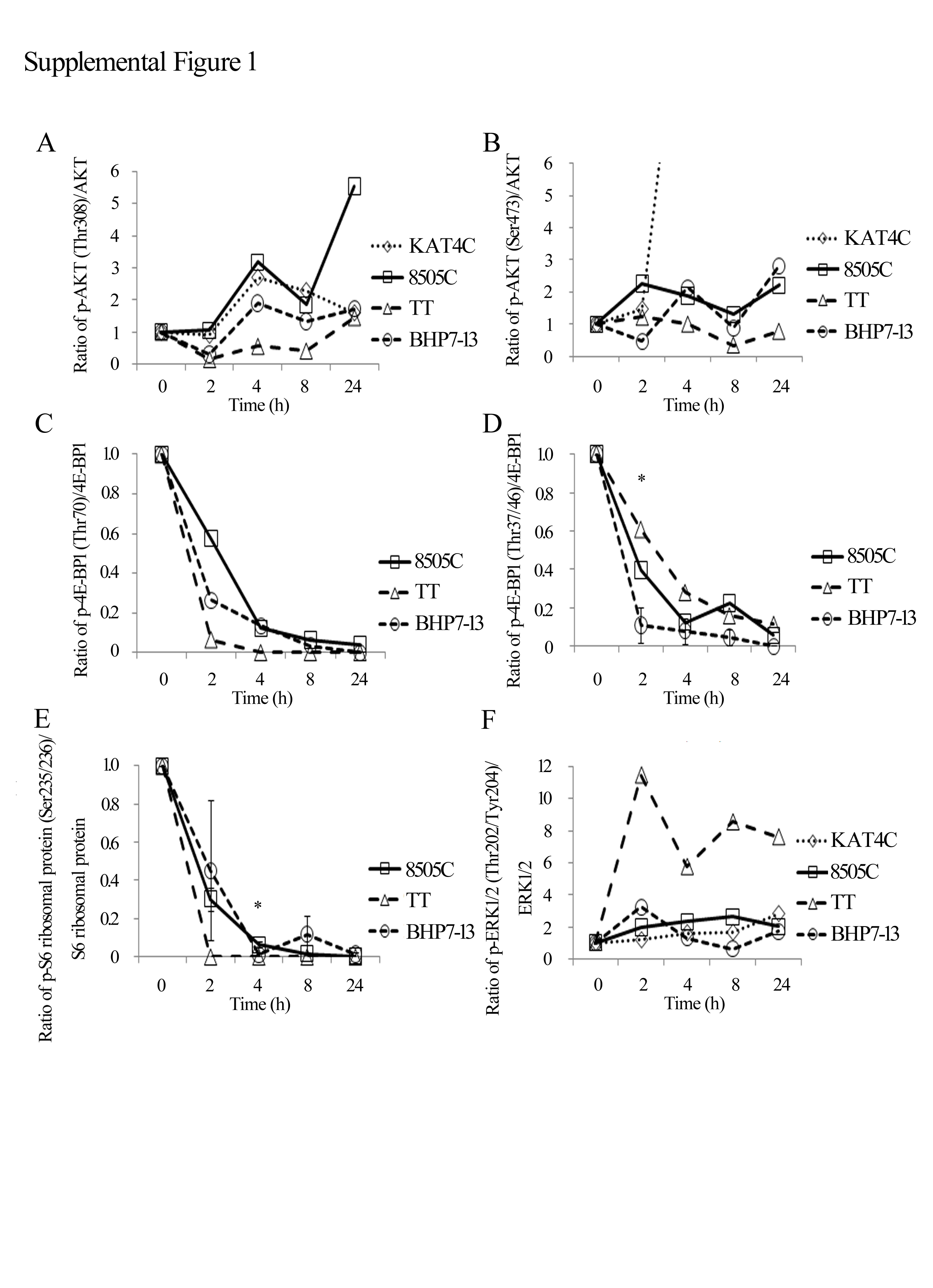

Supplement: Figure S1 — BEZ235 consistently inhibits mTORC1 signaling and activates p-ERK1/2. A, p-AKT (Thr308) was transient reduced by 2 hours in TT and BHP7-13 and increased by 4 hours in 8505C and KAT4C. B, p-AKT (Ser473) was reduced by 2 and 8 hours in TT and BHP7-13 and increased by 2 hours in 8505C and KAT4C. C, p-4E-BP1 (Thr70) was consistently reduced by 2 hours in 3 cell lines. D, p-4E-BP1 (Thr37/46) was reduced by 2 hours. The extent of repression is statistically significant from 2 to 24 hours in BHP7-13 compared with baseline level. E, p-S6 ribosomal protein (Ser235/236) was decreased by 2 hours. The inhibition was significant in 8505C and TT from 4 to 24 hours compared with basal level. F, p-ERK1/2 (Thr202/Tyr204) was activated by 2 to 4 hours in four cell lines. * denoted P<0.02. (TIF) [file pone.0046726.s003.tif]

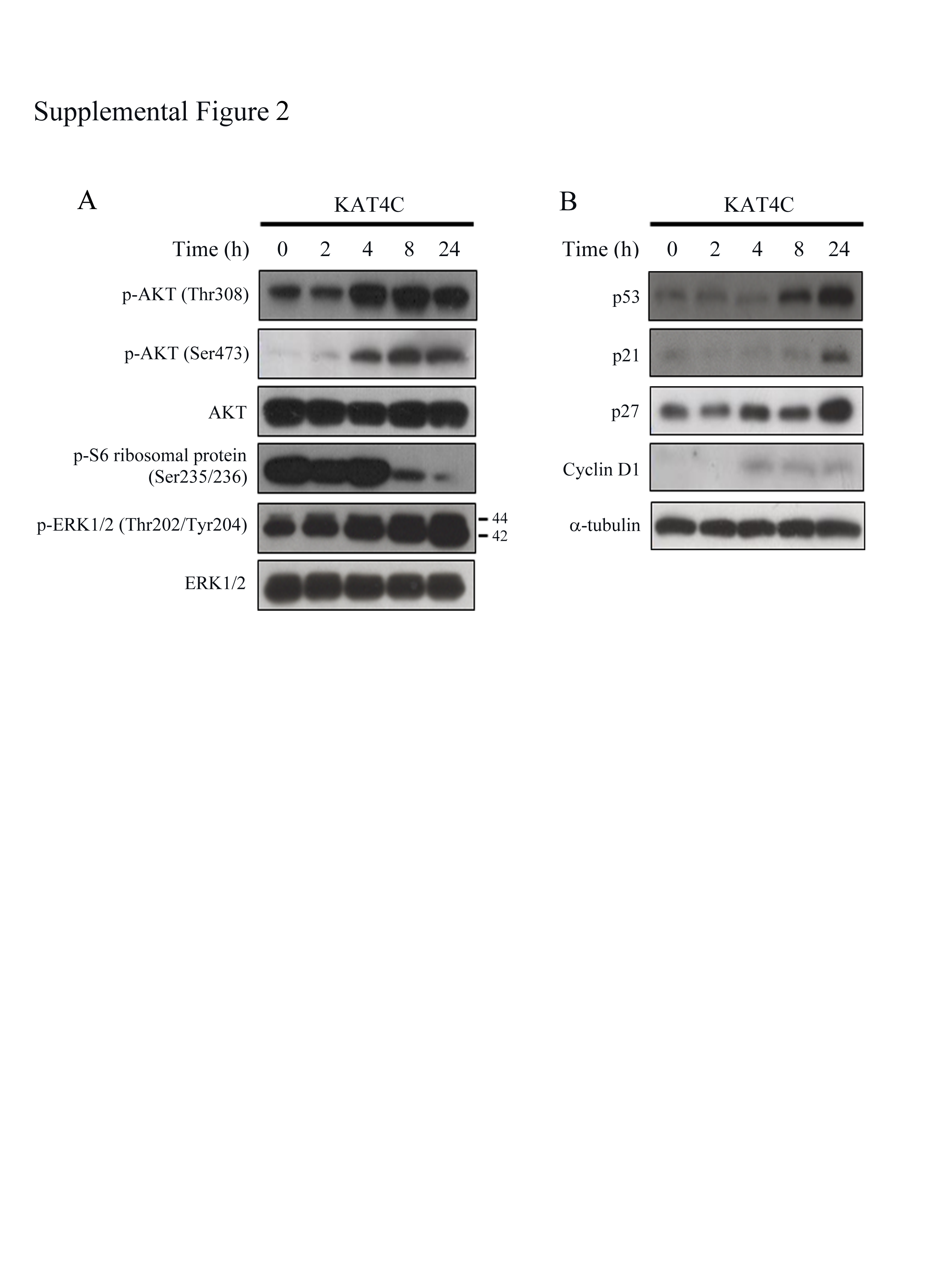

Supplement: Figure S2 — BEZ235 inhibits p-S6 ribosomal protein (Ser235/236) and activates p-ERK1/2 and p27 in KAT4C. A, p-AKT (Thr308), p-AKT (Ser473) and p-ERK1/2 (Thr202/Tyr204) was increased by 4 hours and persisted for more than 24 hours. p-S6 ribosomal protein (Ser235/236) was reduced from 8 hours through over 24 hours. B, p27 was increased by 24 hours. p53, p21 and cyclin D1 were increased by 4 to 24 hours. (TIF) [file pone.0046726.s004.tif]

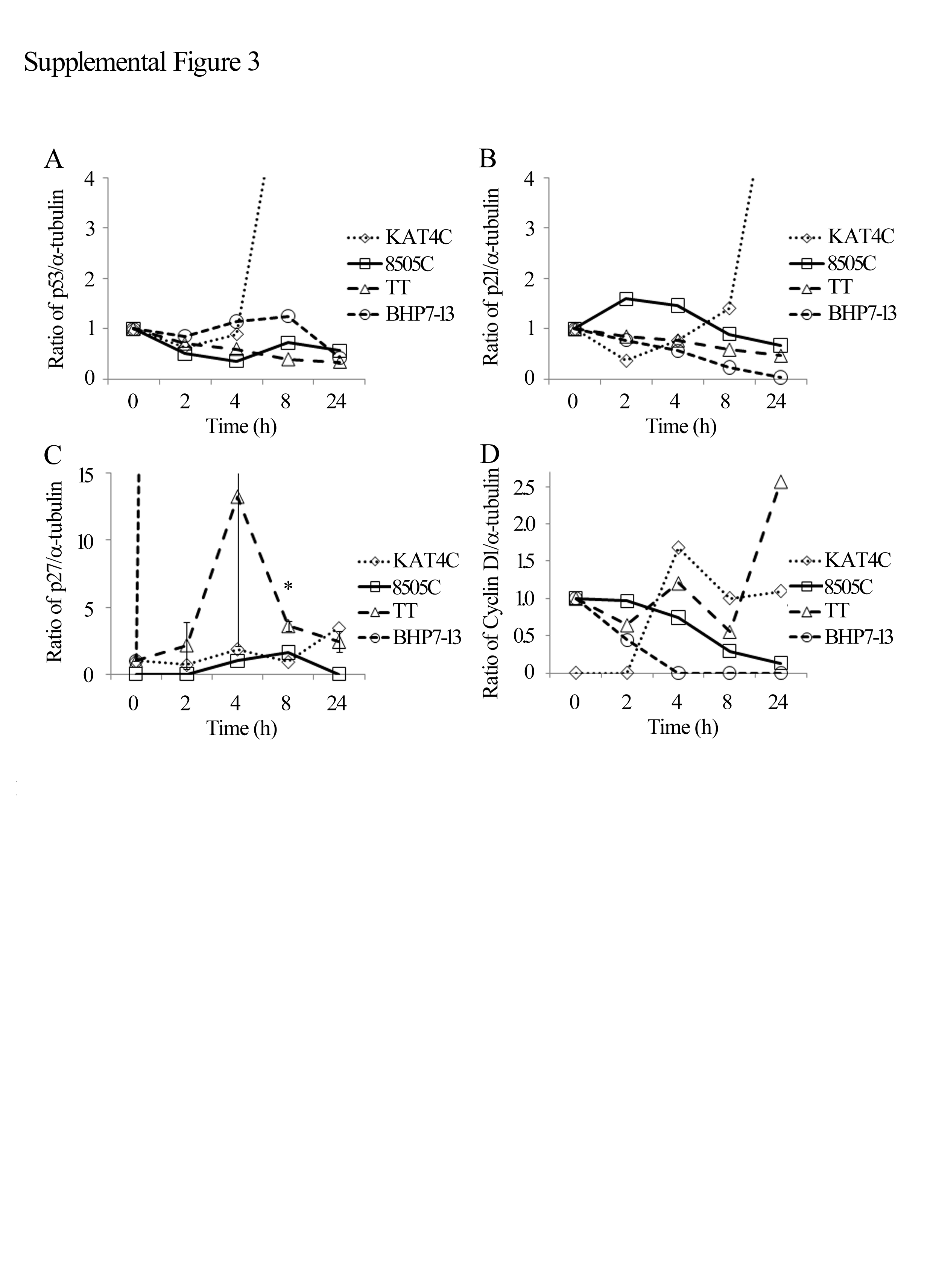

Supplement: Figure S3 — BEZ235 activates p27 expression in thyroid cancer cell lines. A, p53was decreased by 2 to 4 hours in 8505C and TT and increased in KAT4C by 8 hours. B, p21 was repressed in TT and BHP7-13 and increased in KAT4C by 8 hour. C, p27 was increased in all cell lines. The elevation of p27 achieved statistical significance at 8 hour in TT. There was no detectable signal in basal 8505C cells, therefore the reference data was at 4 h. D, Cyclin D1 was gradually decreased in 8505C and BHP7-13 and increased at certain time points in KAT4C and TT. There was no detectable signal in untreated KAT4C, therefore the reference data was 8 h. * denoted P = 0.019 (t-test). (TIF) [file pone.0046726.s005.tif]

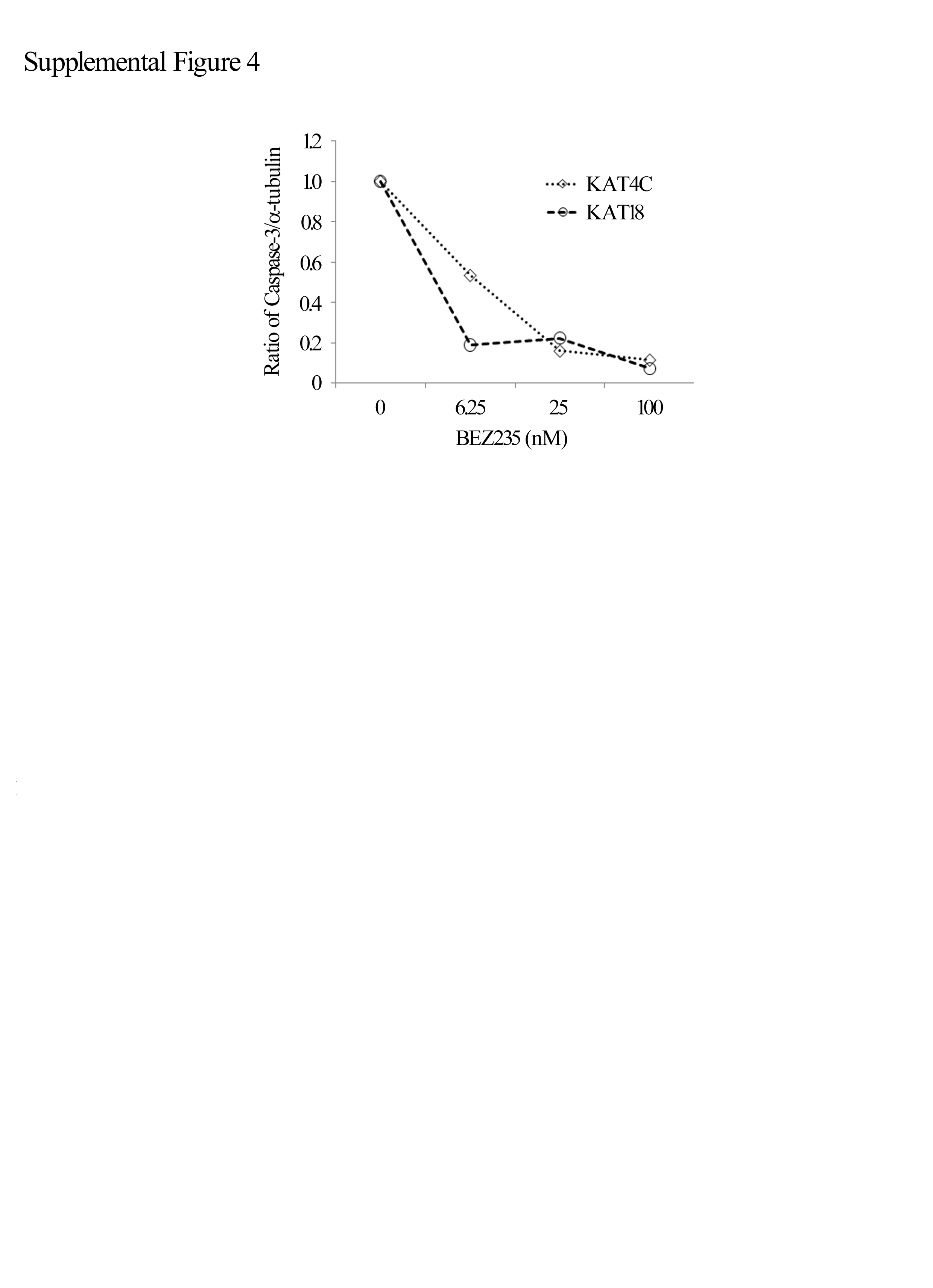

Supplement: Figure S4 — BEZ235 degrades caspase-3 in vitro. Quantification of immunoblot showed caspase-3 was decreased in KAT4C and KAT18 at 72 hours at doses ranged from 6.25 to 100 nmol/L. (TIF) [file pone.0046726.s006.tif]

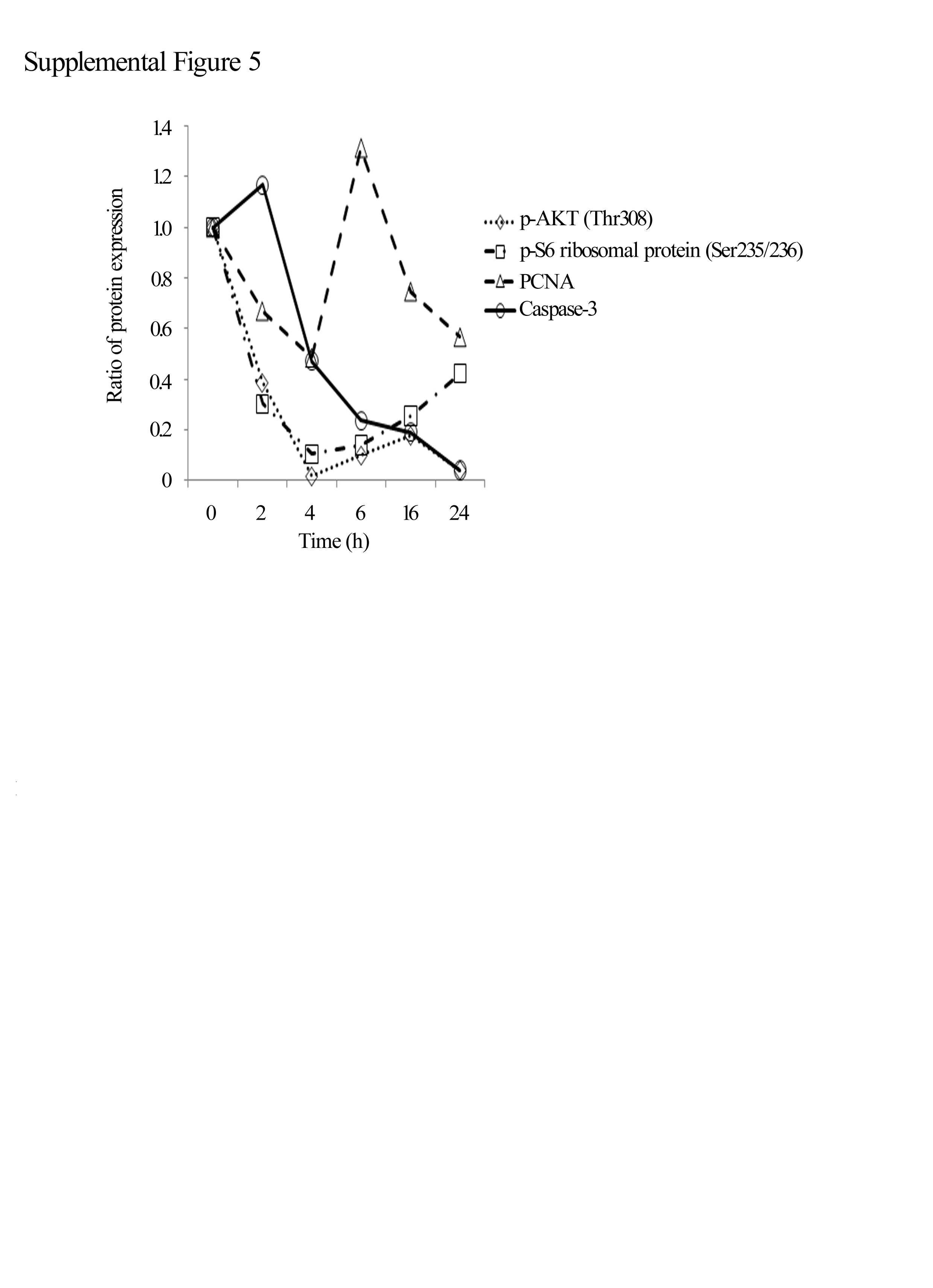

Supplement: Figure S5 — BEZ235 represses the expression of p-AKT, p-S6 ribosomal protein and capase-3 in vivo. The effects of BEZ235 on p-AKT, p-S6 ribosomal protein, PCNA and capase-3 in vivo. Quantification of immunoblot showed BEZ235 greatly repressed p-AKT (Thr308), p-S6 ribosomal protein (Ser235/236) and caspase-3 by 2 to 4 hours with durable effects. PCNA was slightly reduced at an early time point. (TIF) [file pone.0046726.s007.tif]

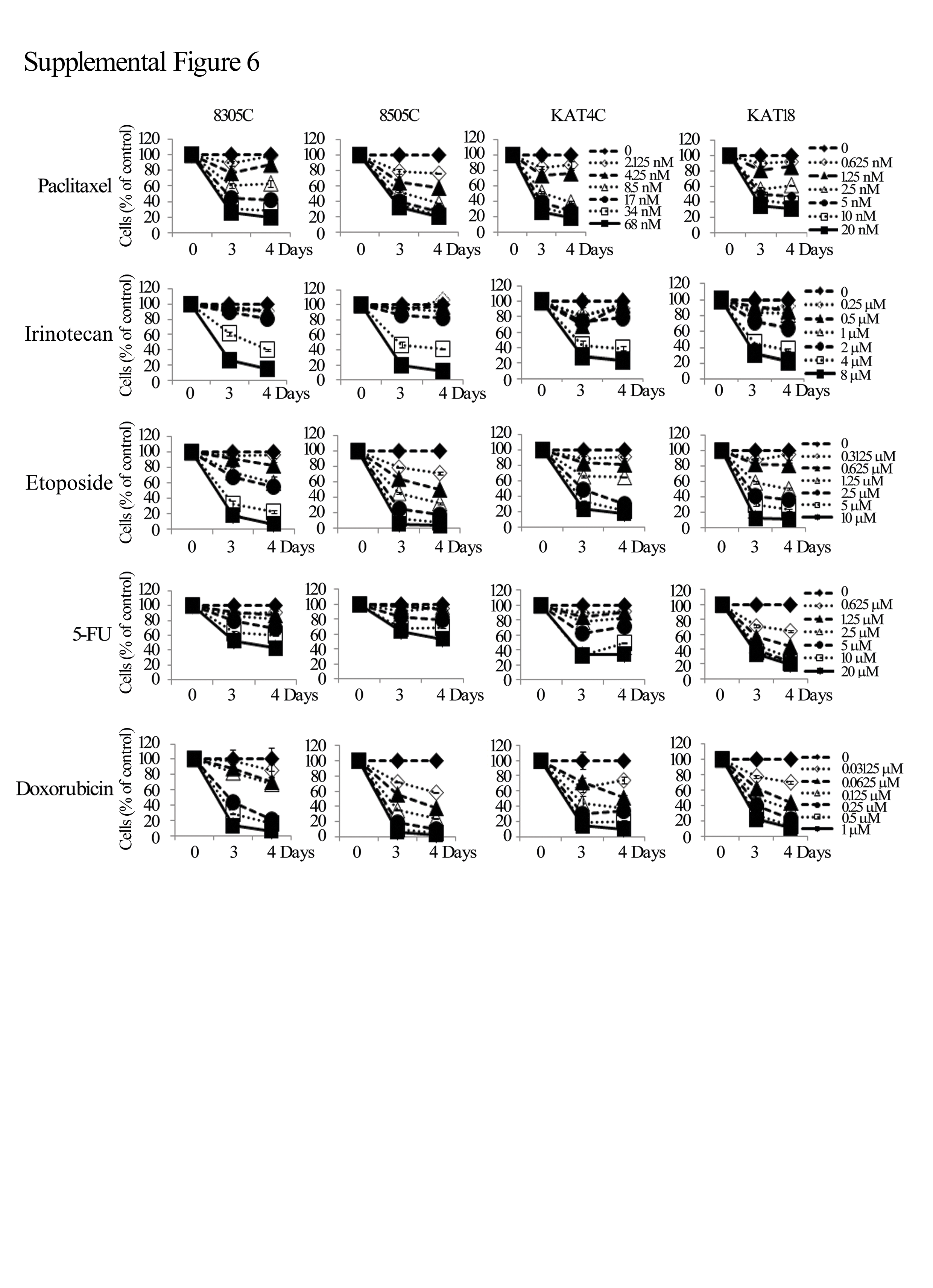

Supplement: Figure S6 — Five chemotherapeutic agents induce dose and time dependent cytotoxicity in 4 anaplastic thyroid cancer cell lines. Dose-response curves were obtained on day 3 and 4 from cells treated with serial dilutions of chemotherapeutic agents (paclitaxel, irinotecan, etoposide, 5-FU, doxorubicin) for a 4-day course on ATC cell lines using LDH assays. All drugs demonstrated dose and time dependent cytotoxicity in four cell lines. (TIF) [file pone.0046726.s008.tif]
